# Mapping the network structure of anxiety, depression, and sleep symptoms in patients with polycystic ovary syndrome

**DOI:** 10.3389/fpsyt.2026.1738355

**Published:** 2026-01-30

**Authors:** Shaojing Li, Yuhong Zhang, Xiangze Tang, Meilian Deng, Hong Luo, Yun Chen

**Affiliations:** Department of Obstetrics and Gynecology, Guangdong Provincial Key Laboratory of Major Obstetric Diseases, Guangdong Provincial Clinical Research Center for Obstetrics and Gynecology, Guangdong-Hong Kong-Macao Greater Bay Area Higher Education Joint Laboratory of Maternal-Fetal Medicine, The Third Affiliated Hospital, Guangzhou Medical University, Guangzhou, China

**Keywords:** anxiety, depression, network analysis, polycystic ovary syndrome, sleep problems

## Abstract

**Background:**

Patients with polycystic ovary syndrome (PCOS) often experience anxiety, depression, and sleep problems in addition to endocrine and metabolic abnormalities, which may adversely affect their quality of life and disease progression. However, research on the co-occurrence patterns and interconnections among these psychological symptoms in PCOS remains limited. This study applied network analysis to explore the relationships among anxiety, depression, and sleep problems in women with PCOS, aiming to identify key symptom nodes and provide evidence for targeted psychological interventions.

**Methods:**

This retrospective study included 1,068 patients with PCOS. Anxiety, depression, and sleep problems were assessed using the Generalized Anxiety Disorder Scale (GAD-7), Patient Health Questionnaire (PHQ-9), and Pittsburgh Sleep Quality Index (PSQI). Symptom networks were estimated using the Least Absolute Shrinkage and Selection Operator (LASSO) and Extended Bayesian Information Criterion (EBIC) in R software. Central and bridge symptoms were identified using expected influence (EI) and bridge expected influence (BEI). Network stability and accuracy were evaluated through bootstrap methods. The Network Comparison Test (NCT) was applied to analyze network differences across subgroups that vary by marital status and weight status.

**Results:**

Network analysis revealed that the strongest edge was the connection between “Daytime dysfunction” and “Low energy” (PSQI7-PHQ4), spanning different symptom communities. Another edge of high intensity was observed between “Worthlessness” and “Suicidal ideation” (PHQ6-PHQ9). “Trouble relaxing” (GAD4) and “Sad mood” (PHQ2) exhibited the highest EI values within the network. Meanwhile, “Daytime dysfunction” (PSQI7) and “Low energy” (PHQ4) showed the highest BEI values. Network comparison analysis revealed no significant differences across marital and weight status subgroups.

**Conclusion:**

This study is the first to explore the symptom interrelationships among anxiety, depression, and sleep problems in PCOS patients. Targeting these central symptoms (e.g., trouble relaxing, sad mood) and bridging symptoms (e.g., daytime dysfunction, low energy) may more effectively alleviate patients’ overall psychological issues and potentially interrupt the spread of comorbid psychiatric conditions. The findings of this study can inform the development of personalized mental health management strategies for patients with PCOS.

## 1 Introduction

Polycystic ovary syndrome (PCOS) is a common endocrine-metabolic disorder affecting 5%-18% of women, with impacts throughout their entire life cycle, including adolescence, the reproductive years, and the postmenopausal phase (1). According to the Rotterdam criteria, PCOS is diagnosed by the presence of at least two of the following features: polycystic ovarian morphology, ovulatory dysfunction, and clinical and/or biochemical hyperandrogenism (2). Its clinical presentation exhibit high heterogeneity, including menstrual irregularities, insulin resistance, obesity, acne, hirsutism, and infertility. In addition to reproductive and metabolic disturbances, accumulating evidence indicates that individuals with PCOS also commonly suffer from psychological issues (3). Among these, anxiety and depression are the most prevalent emotional disorders (4).

The Global Burden of Disease (GBD) study states that anxiety and depression have grown to be significant global mental health issues (5), significantly increasing the economic burden of disease. A meta-analysis revealed that the prevalence of anxiety and depression among PCOS patients was 37% and 42%, respectively (6). Compared to healthy women, PCOS patients face a 6-fold increased risk of moderate-to-severe anxiety symptoms and a 4-fold increased risk of moderate-to-severe depression symptoms (7). Concurrently, sleep problems are also highly prevalent in this population (8). The Diagnostic and Statistical Manual of Mental Disorders, Fifth Edition (DSM-5), recognizes sleep disorders as a distinct category of mental illness (9). A recent study has reported that more than half of women with PCOS experience varying degrees of sleep disturbance, with nearly one-third suffering from chronic sleep impairment (10). These psychological and sleep problems not only increase psychological distress and compromise treatment effectiveness, but also significantly raise healthcare expenses and elevate long-term risks, including suicidality (11–14).

Notably, anxiety, depression, and sleep problems frequently co-occur in women with PCOS (10, 15), suggesting that these symptoms may not arise independently but may form a more tightly intertwined association within a specific disease context. PCOS is characterized by hyperandrogenism, insulin resistance, and chronic low-grade inflammation, all of which may influence neuroendocrine regulation, hypothalamic-pituitary-adrenal (HPA) axis function, and physiological processes related to emotion and sleep, thereby increasing vulnerability to emotional dysregulation and sleep disturbance (16–21). Additionally, body image concerns such as obesity and acne, along with infertility stress, can easily lead to psychological issues in patients, including anxiety, worry, and low self-esteem (22, 23). Under the combined influence of biological dysregulation and psychosocial stress, anxiety and depressive symptoms may be more readily activated and become more tightly linked with sleep problems. Empirical studies have shown that women with PCOS who report anxiety symptoms tend to have shorter sleep duration (24), whereas those with sleep disorders exhibit significantly elevated risks of anxiety and depression (25). These findings suggest that, in the context of PCOS, the interactions among anxiety, depression, and sleep problems may be structurally reinforced, with certain symptoms playing a more central role in sustaining overall psychological distress.

However, existing research primarily employs traditional assessment methods based on total scale score models, focus on evaluating overall severity and its correlations with other variables, yet overlook the interrelationships between symptoms and their heterogeneity (26, 27). Recently, network analysis, a novel emerging data-driven approach, has provided new insights into the structure of psychopathology and the interactions between symptoms (28). This method builds upon Borsboom’s (2017) theoretical framework (29), which views mental disorders as emergent phenomena resulting from direct interactions between symptoms. Each symptom is represented as a node within the network, and correlations between symptoms form edges of the network, enabling the visual revelation of the interrelations and underlying connections between the symptoms. Within this framework, symptoms that occupy more central positions in the network are considered more influential in maintaining the overall symptom structure, whereas bridge symptoms may link different symptom clusters and contribute to comorbidity. Identifying such symptoms may help elucidate patterns of symptom co-occurrence and offer potential directions for more targeted psychological assessment and management (30, 31).

Currently, network analysis has been widely employed to explore the intricate interrelations among depression, anxiety, and sleep problems, revealing both shared and distinct network characteristics across different populations (32, 33). These findings suggest that symptom interrelations may vary across populations with distinct clinical and psychosocial profiles. Although the growing application of network analysis in psychiatric research (34), studies focusing on women with PCOS remain limited.

Given the complex biopsychosocial features of PCOS, the present study will employ the network analysis approach to construct a network model of anxiety, depression, and sleep problems to explore the interrelationships among psychological symptoms. The study aims to explore fine-grained associations between these three types of symptoms and identify the key central and bridging symptoms. Additionally, the Network Comparison Test (NCT) is used to analyze whether these connections vary based on marital status and clinical features (35). The findings are expected to better assist clinicians in developing more precise mental health management strategies, thereby improving PCOS patients’ mental health and quality of life.

## 2 Materials and methods

### 2.1 Study design and participants

This retrospective observational study was designed and reported in accordance with the Strengthening the Reporting of Observational Studies in Epidemiology (STROBE) guidelines (36). The study included women diagnosed with PCOS who had established specialized records at the Gynecology Outpatient Department of the Third Affiliated Hospital of Guangzhou Medical University between January 2022 and December 2024. Ethical approval was obtained from the Ethics Committee of the Third Affiliated Hospital of Guangzhou Medical University, and all study procedures adhered to the principles outlined in the Declaration of Helsinki. Because this study utilized retrospective data extracted from medical records and an electronic database, anonymizing all data, the Ethics Committee approved a waiver of informed consent.

We screened electronic databases of all patients who established specialized records at the Gynecology Outpatient Department between January 2022 and December 2024 to identify cases diagnosed with PCOS. A trained investigator independently reviewed the identified cases to confirm whether they met the diagnostic criteria for PCOS according to the 2003 Rotterdam consensus (37). Inclusion criteria were (1) aged 18–40 years; (2) meeting at least two of the three Rotterdam criteria: (a) oligo-ovulation or anovulation, (b) clinical and/or biochemical hyperandrogenism, or (c) polycystic ovarian morphology detected by ultrasonography. Exclusion criteria were (1) pregnant or lactating women; (2) perimenopausal or early menopausal women; (3) history of confirmed psychiatric or neurological disorders; (4) major life events within the past 6 months; (5) incomplete completion of psychological or sleep questionnaires; and (6) missing data exceeding 20% or incomplete clinical records. Following the application of these strict criteria, 1,068 women with PCOS were included in the final analysis.

### 2.2 Measurements

#### 2.2.1 Generalized anxiety disorder scale

The Generalized Anxiety Disorder Scale (GAD-7) was used to assess the severity of anxiety symptoms (38). The GAD-7 has seven items, and each item is rated from 0 (“not at all”) to 3 (“nearly every day”), yielding a total score ranging from 0 to 21. Higher scores indicate greater symptom severity, and a total score ≥ 5 is considered indicative of mild anxiety. The GAD-7 has demonstrated satisfactory reliability and validity in Chinese populations and is widely used as a brief anxiety screening tool (39). In the present study, the Cronbach’s α coefficient for the GAD-7 was 0.89, reflecting good internal consistency.

#### 2.2.2 Patient health questionnaire

The Patient Health Questionnaire (PHQ-9) was used to evaluate the severity of symptoms of depression (40). It is a widely recognized and reliable screening tool for depression. The PHQ-9 has nine items, and each item is rated from 0 (“not at all”) to 3 (“nearly every day”), yielding a total score ranging from 0 to 27. Higher scores indicate greater symptom severity, and a total score≥5 was categorized as mild depression. The PHQ-9 has been extensively validated in Chinese populations (41), and in the current study, its Cronbach’s α coefficient was 0.84. Previous studies have mainly focused on sleep duration and insomnia symptoms (e.g., difficulty falling asleep or staying asleep), with limited attention to sleep dimensions (e.g., sleep efficiency, daytime dysfunction) (42). Therefore, we excluded the third item (Sleep) from this scale and employed the Pittsburgh Sleep Quality Index (PSQI) to assess the complexity of sleep problems comprehensively.

#### 2.2.3 Pittsburgh sleep quality index

The Pittsburgh Sleep Quality Index (PSQI) is a widely used instrument for assessing sleep quality (43). It consists of 19 items encompassing seven dimensions: subjective sleep quality, sleep latency, sleep duration, sleep efficiency, sleep disturbances, use of sleep medication, and daytime dysfunction. Each dimension is scored on a 0–3 scale, and the total score of PSQI is 21. Higher scores indicate a more severe sleep problem. A PSQI total score of more than 7 generally indicates poor sleep quality (44), suggesting the presence of sleep problems.

#### 2.2.4 Clinical features assessment

Clinical features were assessed based on standard diagnostic criteria (45). Hirsutism was evaluated using the modified Ferriman–Gallwey (mFG) score, with a total score of ≥4 indicating hirsutism in Chinese women. Acne was rated according to the Chinese Acne Treatment Guidelines (2019), and participants with moderate or more severe acne were considered acne-positive. Acanthosis nigricans was identified by hyperpigmented, velvety, and thickened skin in typical locations, including the posterior and lateral neck, axillae, inframammary region, and groin. Infertility was defined as the inability to conceive after 12 months of unprotected intercourse. Overweight and obesity were determined according to Chinese adult BMI criteria, with BMI ≥24 kg/m² classified as overweight/obese.

### 2.3 Statistical analysis

We used SPSS Statistics (Version 25.0) to perform descriptive statistics, analyzing demographic information and scores across various scales. Subsequently, we employed R (Version 4.4.1) to construct a comorbidity network for anxiety, depression, and sleep problems. Before building the network, we assessed item redundancy using the correlation matrix method (i.e., correlation coefficients r > 0.7 were considered potentially redundant) (46).

#### 2.3.1 Network estimation

We employed the bootnet and qgraph R packages to construct a regularized partial correlation network model (47). Every node represents a variable or symptom, and edges represent pairwise associations between nodes in the network. Blue and red lines represent positive and negative correlations, respectively. The correlation’s strength is shown by line thickness and color saturation. In order to obtain a sparse and interpretable network, this study employed the Least Absolute Shrinkage and Selection Operator (LASSO) (48)combined with the Extended Bayesian Information Criterion (EBIC) (49). Spearman’s rank correlation coefficient was used for correlation calculations because of the skewed distribution of psychological symptom scores (50). Additionally, in accordance with the conservative default suggested for psychological network analysis, the EBIC tuning value (γ) was set at 0.5 to improve both interpretability and resilience (49).

Expected influence (EI) was used as the metric for node centrality in this study. Compared to traditional centrality measures (such as strength centrality), it is more suitable for symptom networks with both positive and negative edges. EI is the sum of the absolute values of the edge weights connecting the node to all other nodes. Higher values of EI reflect greater importance within the network and are considered as core symptoms (51). Bridge symptoms are identified by computing the bridge expected influence (BEI). A higher BEI value indicates a stronger bridging role in the activation and propagation between symptom clusters (52). EI and BEI were computed using the R packages qgraph and networktools.

Additionally, the R package mgm was used to estimate node predictability (i.e., R^2^) (53), which represents the proportion of variance of a node explained by its neighboring nodes. Symptoms with high predictability symptoms indicate that they can be intervened on indirectly through adjacent symptoms (54). In contrast, symptoms with low predictability might require direct intervention or an examination of external factors beyond the network.

#### 2.3.2 Network stability and accuracy

To evaluate the network’s accuracy and stability, we used the R program Bootnet. First, we used a nonparametric bootstrap approach to assess the accuracy of edge weights by calculating 95% confidence intervals (CIs) (55). Narrower confidence ranges indicate more stable and reliable edge weight estimates. Second, a case-dropping bootstrap approach that measures the correlation stability coefficient (CS-C) was used to investigate the stability of centrality indices (EI and BEI). A CS-C value should not fall below 0.25; values exceeding 0.50 indicate robust and interpretable network structures (56). Finally, we conducted significance tests on the EI, BEI, and edge weights across different nodes and examined the significant differences in centrality metrics within the network (48). All analyses were performed with 1000 bootstrap iterations.

#### 2.3.3 Network comparison

To explore differences in the network structures of anxiety, depression, and sleep problems among women with PCOS, we analyzed the data based on network comparisons were conducted across subgroups defined by clinical features (including infertility, hirsutism, acne, acanthosis nigricans, and overweight/obese status). The R package Network Comparison Test (NCT) was employed to conduct 1000 permutation tests, evaluating differences in global strength and network structure across subgroups (35). A two-tailed p-value < 0.05 was considered statistically significant. Subsequently, we employed the Bonferroni-Holm correction to adjust for multiple comparisons and evaluate edge strength differences.

## 3 Results

### 3.1 Characteristics of the study sample

This study included 1,068 patients with PCOS, aged 18–40 years (mean 27.77 ± 4.44). Within this population, 640 (59.9%) were married, 591 (55.3%) had fertility desires, and 334 (31.3%) were overweight/obese (BMI≥24). Notably, 503 participants (47.1%) displayed anxiety symptoms (GAD-7 score ≥ 5), 440 (41.2%) exhibited depressive symptoms (PHQ-9 score ≥ 5), and 288 (27.0%) reported sleep problems (PSQI global score > 7). Additional information is shown in **Table 1**. The mean, standard deviation (SD), expected influence (EI), bridge expected influence (BEI), and predictability of all items across the scales are presented in **Supplementary Table S1**. Furthermore, no redundant item was detected between any items within this network (i.e., correlation coefficient showing more than 0.7).

**Table 1:** Demographic characteristics of the study participants (n=1068).

Variables	Mean/N	SD/%
Age (year)	27.77	4.44
Marital status		
Married	640	59.9
Unmarried	428	40.1
BMI(kg/m^2^)		
Underweight (≤18.4)	133	12.5
Normal weight (18.5-23.9)	601	56.2
Overweight/Obesity (≥24)	334	31.3
Fertility desires		
Yes	591	55.3
No	477	44.7
Smoking		
Yes	37	3.5
No	1031	96.5
Alcohol consumption		
Yes	78	7.3
No	990	92.7
Staying up late		
Yes	874	81.8
No	194	18.2
Infertility		
Yes	295	27.6
No	773	72.4
Hirsutism		
Yes	327	30.6
No	741	69.4
Acne		
Yes	394	36.9
No	674	63.1
Acanthosis nigricans		
Yes	209	19.6
No	859	80.4
Anxiety (GAD-7)		
No anxiety (0-4)	565	52.9
With anxiety (5-21)	503	47.1
Depression (PHQ-9)		
No depression (0-4)	628	58.8
With depression (5-27)	440	41.2
Sleep problem (PSQI)		
Normal sleep (0-7)	780	73.0
Have sleep problems (8–21)	288	27.0

### 3.2 Network estimation

**Figure 1:**
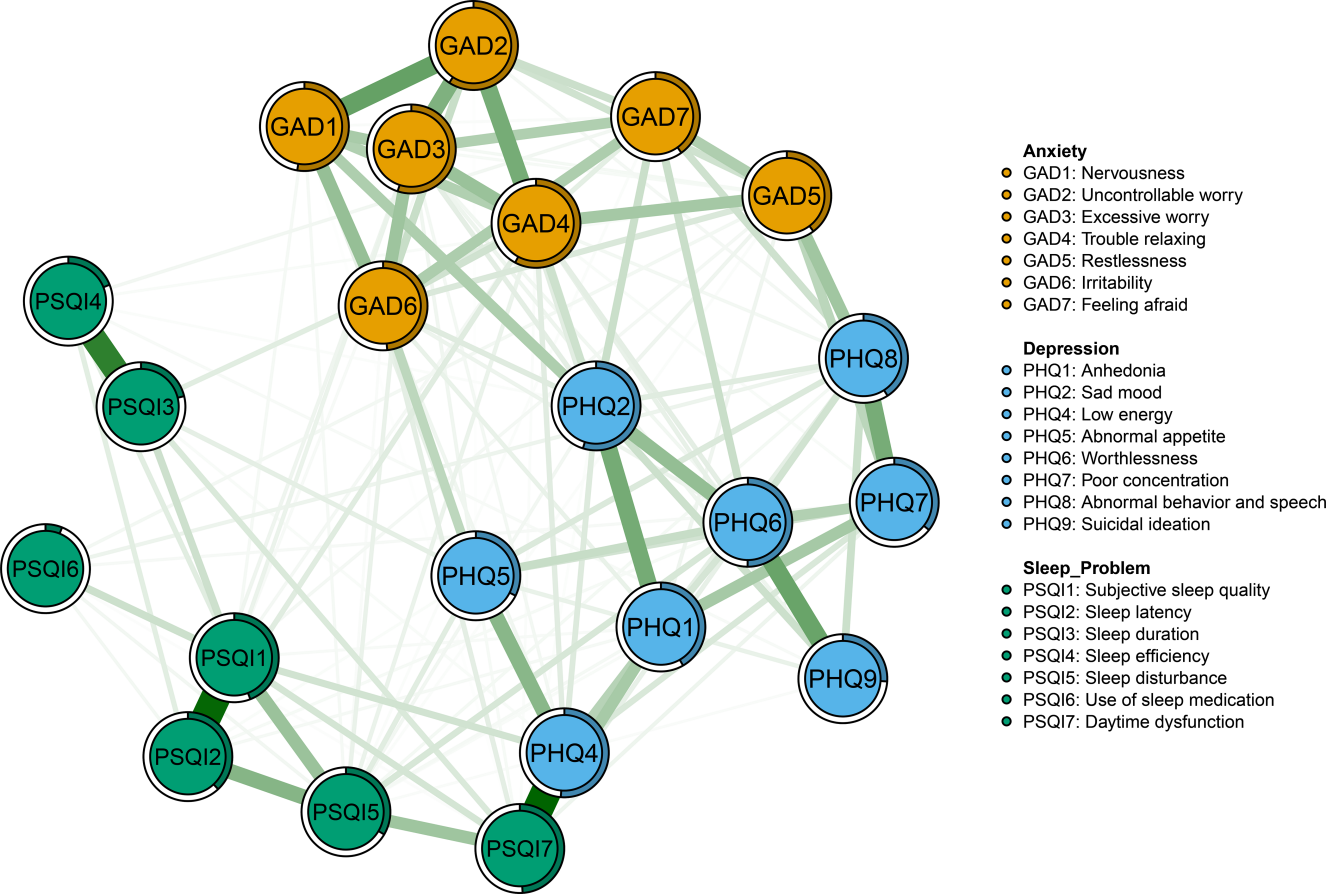
Network structure of anxiety, depression and sleep problems in patients with PCOS.

The anxiety, depression, and sleep problems network model of PCOS patients is depicted in **Figure 1**. Within this network structure, among 253 possible edges, 139 non-zero edges were identified (network density=0.55), comprising 135 positive edges and 4 negative edges. The strongest edge, which links PHQ4 “low energy” in depression and PSQI7 “Daytime dysfunction” in sleep problems, is found across communities (weight=0.403). The remaining firm edges are primarily concentrated within their respective communities. Within the sleep symptom community, the strongest edges were observed between PSQI1 “Subjective sleep quality” and PSQI2 “Sleep latency” (weight=0.396), and between PSQI3 “Sleep duration” and PSQI4 “Sleep efficiency” (weight=0.329). For anxiety symptoms, significant connections included GAD1 “Nervousness”-GAD2 “Uncontrollable worry” (weight=0.244), GAD2 “Uncontrollable worry”-GAD4 “Trouble relaxing” (weight=0.216), and GAD2 “Uncontrollable worry”-GAD3 “Excessive worry” (weight=0.202). Among depressive symptoms, the strongest connections were PHQ6 “Worthlessness”-PHQ9 “Suicidal ideation” (weight=0.238), PHQ1 “Anhedonia”-PHQ2 “Sad mood” (weight=0.218), and PHQ7 “Poor concentration”-PHQ8 “Abnormal behavior and speech” (weight=0.215). For detailed weights of all edges, please refer to **Supplementary Table S2** in the **Supplementary Materials**.

In addition, the predictability of each node is represented by the outer rings surrounding the nodes in **Figure 1**. The average predictability across all network nodes is 0.42, indicating that 42% of node variance can be explained by their neighboring nodes. Among them, “Excessive worry” (GAD3) and “Trouble relaxing” (GAD4) show high predictability, whereas “Use of sleep medication” (PSQI6) had the lowest predictability.

### 3.3 Central symptoms and bridge symptoms

**Figure 2:**
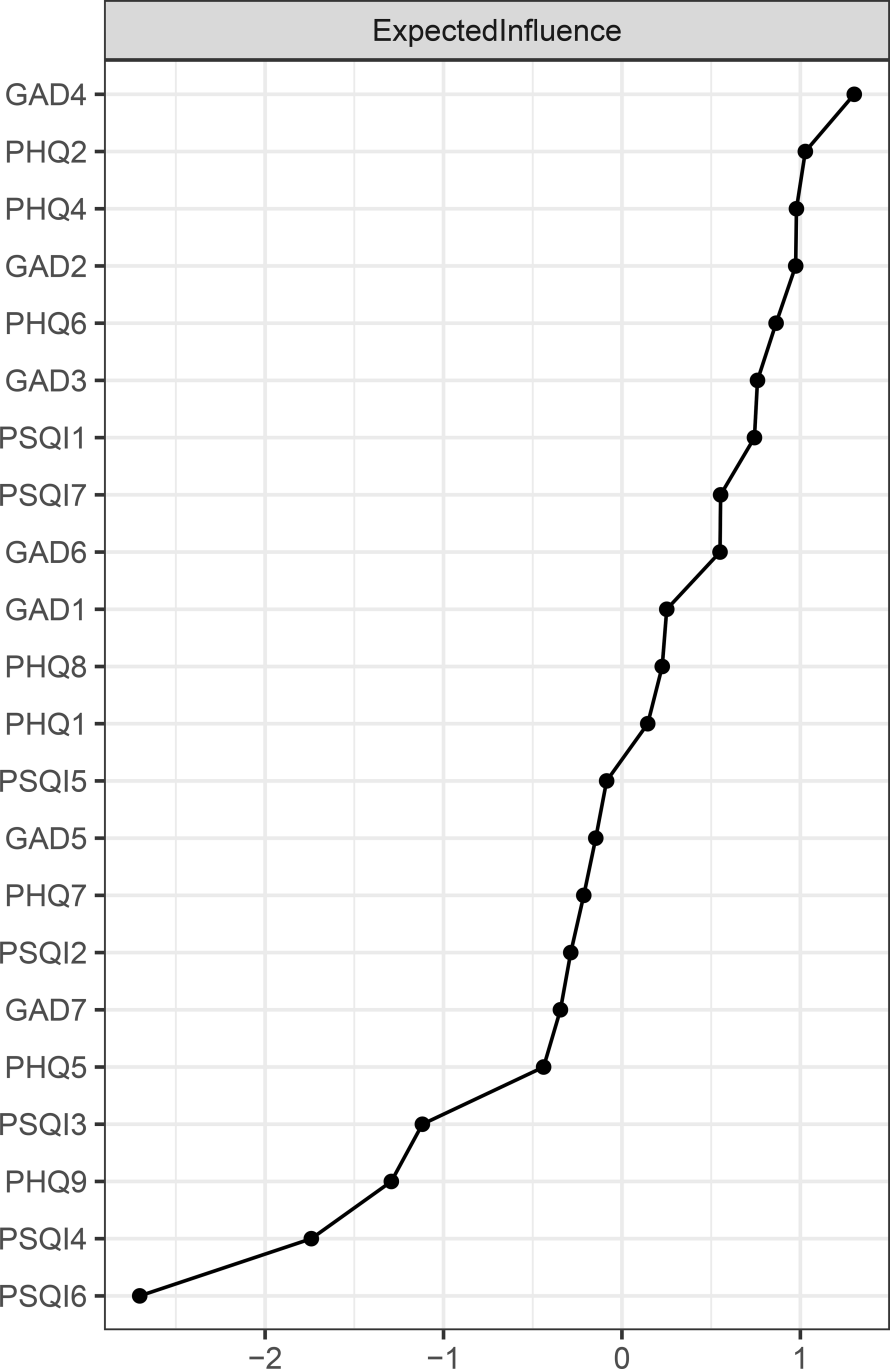
Standardized expected influence (EI) centrality of network structure of anxiety, depression and sleep problems in patients with PCOS (z-scores). For clarity, the nodes with the highest EI values are labeled as follows: GAD4=trouble relaxing; PHQ2=sad mood; PHQ4=low energy. Full item wording is provided in **Supplementary Table S1**.

**Figure 3:**
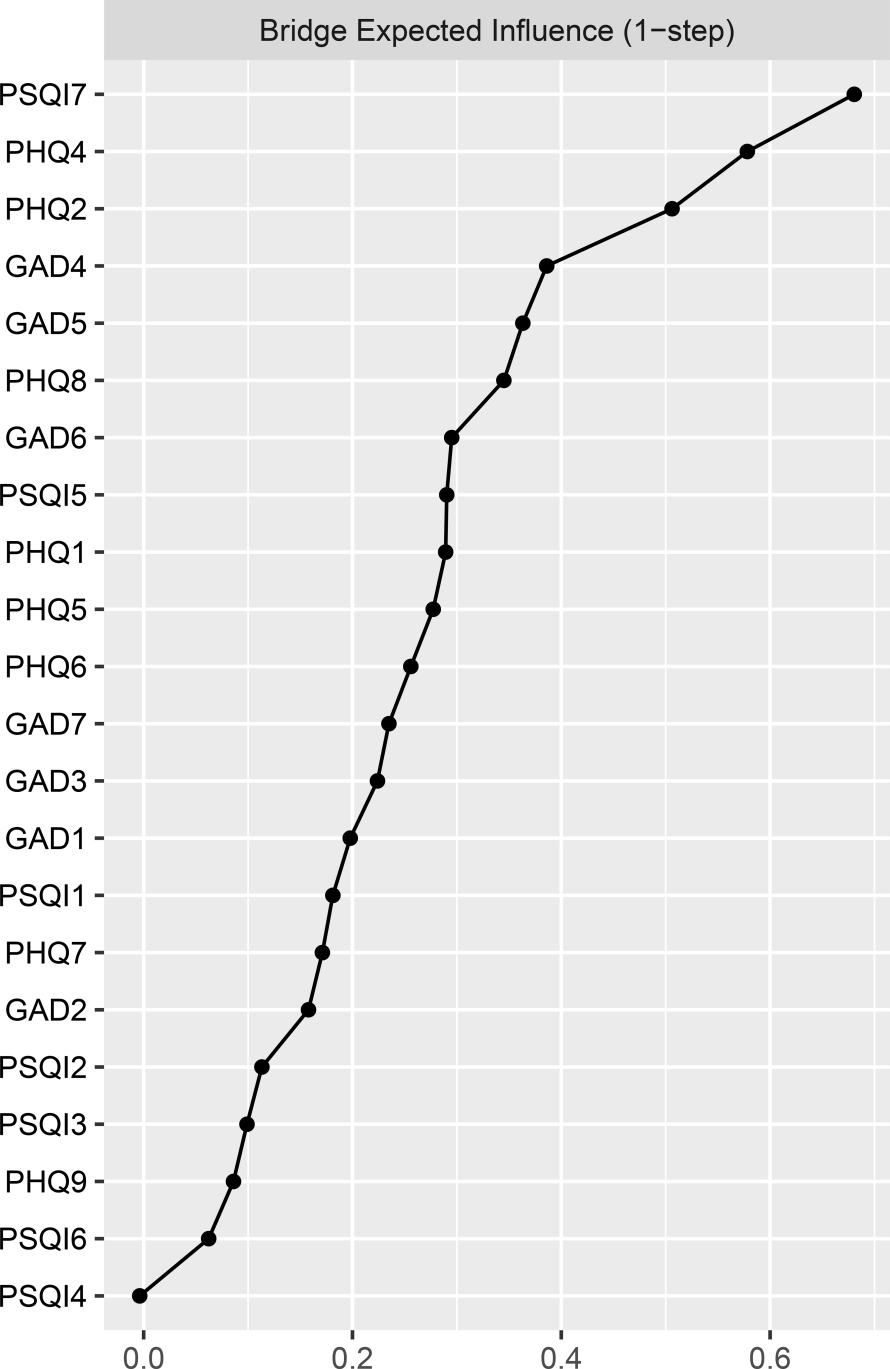
Standardized bridge expected influence (BEI) centrality of network structure of anxiety, depression and sleep problems in patients with PCOS (z-scores). For clarity, the nodes with the highest BEI values are labeled as follows: PSQI7=daytime dysfunction; PHQ4=low energy; PHQ2=sad mood. Full item wording is provided in **Supplementary Table S1**.

The EI results for each node are shown in **Figure 2**. “Trouble relaxing” (GAD4) and “Sad mood” (PHQ2) exhibit the highest expected impact values, followed by “Low energy” (PHQ4), indicating that these three symptoms are the most influential and occupy a central position in the current network. The BEI results for each node are shown in **Figure 3**. “Daytime dysfunction” (PSQI7), “Low energy” (PHQ4), and “Sad mood” (PHQ2) had the highest bridge expected influence. These symptoms serve as crucial bridge nodes linking anxiety, depression, and sleep problems.

### 3.4 Network accuracy and stability

The findings of robustness analyses were as follows. First, nonparametric bootstrapping yielded relatively narrow 95% confidence intervals for edge weights, suggesting precise estimates. Second, the correlation stability coefficient (CS-C) for both expected influence and bridge expected influence reached 0.75, which exceeds the recommended threshold of 0.50. The centrality metrics exhibit strong stability. Finally, bootstrapped difference tests confirmed that the strongest edges (e.g., PHQ4–PSQI7, PSQI1–PSQI2) and the influence values of core (GAD4, PHQ2) and bridge (PSQI7, PHQ4) nodes were significantly greater than those of most others (**Supplementary Figure S1**).

### 3.5 Comparisons based on marital and weight status

Network comparison results revealed invariance in network structure or overall strength between subgroups defined by clinical features (**Supplementary Figures S2-S6**). Regarding global strength, no significant differences were observed between the non-overweight and overweight/obese groups (9.42 vs. 9.07, *P*=0.383), infertility and without infertility groups (9.25 vs. 9.28, *P*=0.950), hirsutism and without hirsutism groups (9.61 vs. 9.30, *P*=0.419), acne and without acne groups (9.15 vs. 9.10, *P*=0.882), or acanthosis nigricans and without acanthosis nigricans groups (9.04 vs. 9.35, *P*=0.531). Similarly, in network structure comparisons, no significant differences were found between weight status (M=0.17, *P*=0.546), infertility(M=0.15, *P*=0.873), hirsutism(M=0.18, *P*=0.480), acne(M=0.15, *P*=0.778), or acanthosis nigricans(M=0.20, *P*=0.528) groups. After Bonferroni-Holm correction, all edge weight differences within subgroups remained non-significant (*P*>0.05).

## 4 Discussion

In this study, we used network analysis to investigate the complex connections between anxiety, depression, and sleep problems in PCOS patients. By identifying central and bridge symptoms within the network, we provide reference points for targeted psychological interventions. Notably, it is the first study to examine psychological symptoms in PCOS patients using network analysis. The findings only offer initial insights, and some discoveries require further validation in future studies.

### 4.1 The fine-grained relationships between anxiety, depression and sleep problems

The symptom network in this study reveals the tightly intertwined relationship among anxiety, depression, and sleep problems. This pattern aligns with findings from other populations, further supporting the existence of a stable associative pattern among these three psychiatric symptoms (57, 58).

Distinct from most network studies in which the strongest associations typically occur within the same symptom community, the present study identified the strongest edge across communities, linking “daytime dysfunction” in sleep problems with “low energy” in depressive symptoms (PSQI7-PHQ4). Existing research indicates that sleep disorders frequently are often accompanied by daytime functional impairment and low-energy states (20, 59, 60). Physiological evidence suggests that sleep deprivation may disrupt the hypothalamic-pituitary-adrenal (HPA) axis and energy metabolism (61, 62), partly contributing to low-energy experiences. In women with PCOS, disease-related pathophysiological features and persistent psychosocial stress may further accentuate this association, making daytime dysfunction a salient correlate of low energy in this population. This cross-community association suggests that daytime functioning may play an important role in the co-occurrence of sleep problems and depressive symptoms among women with PCOS. The next strongest edges were observed within the sleep problems community, indicating tight associations different specific sleep dimensions. These findings suggest that sleep disorders in women with polycystic ovary syndrome rarely occur in isolation (63, 64). Clinical management should focus on the comorbidity and mutual reinforcement of multiple sleep issues, rather than targeting interventions at individual symptoms alone.

Notably, the strongest association within the depressive symptom network was identified between “Suicidal ideation” (PHQ9) and “Worthlessness” (PHQ7). This finding has been relatively underreported in previous network analyses, which commonly focused on the relationship between “suicidal ideation” and “Abnormal behavior and speech “ (65, 66). Although the centrality of suicidal ideation was relatively low in our network, it remains a clinically critical high-risk symptom. Research indicates that “Worthlessness” on the PHQ-9 scale is a significant predictor of suicidal ideation among primary care patients (67). Individuals with suicidal ideation commonly exhibit negative self-beliefs (e.g., low self-worth) (68), and that persistent self-deprecation and feelings of failure may intensify hopelessness, thereby fostering suicidal ideation. This psychological pathway might be further amplified in patients with complex and severe PCOS, who often experience body image dissatisfaction, repeated treatment frustration, and reproductive stress (22, 23, 69). Meanwhile, the physiological changes associated with PCOS also may exacerbate emotional dysregulation and feelings of worthlessness (70, 71). Although the specific mechanisms require further validation, this study suggests that clinicians should pay close attention to early signs of worthlessness (e.g., self-blame, feeling useless, or disappointing others) to prevent the escalation toward suicidal ideation.

### 4.2 The central and bridge symptoms between anxiety, depression and sleep problems

Nodes with higher expected impact usually occupy core positions within symptom networks, offering potential targets for psychological interventions. “Trouble relaxing” (GAD4) had the highest centrality value in this study, suggesting that it plays a crucial role in triggering and maintaining symptom networks of anxiety, depression, and sleep problems. As a core symptom of anxiety disorders, trouble relaxing manifests as persistent somatic tension and difficulty achieving relaxation (72). While “Trouble relaxing” has been widely recognized as clinically relevant in psychopathology (73, 74), its prominent centrality in the PCOS patients may be associated with the prevalence of chronic psychological stress and physiological hypervigilance; however, this hypothesis requires further validation. Although network analysis cannot establish direct causality, this pattern can provide useful insights for clinical psychological management. Specifically, interventions targeting “Trouble relaxing”, identified as a core symptom in the network, may represent a potential entry point, with their impact on the overall psychological symptom network in women with PCOS requiring further validation in future studies.

Furthermore, “sad mood” (PHQ2) was the second core symptom and played an important bridging role within the network. This pattern finds support in a network study of general populations from El Salvador and Peru (75), suggesting that the central role of sad mood may not be unique to PCOS. The DSM-5 lists depressed mood as a core criterion for diagnosing depressive disorders (76), primarily manifested as negative emotional experiences such as sadness, fear, and feelings of failure. Consistent with this, a cross-sectional study focusing on PCOS patients found that sad mood was the most common and prominent symptom of depression (77). The chronic disease burden, psychological stress, and inadequate medical support associated with PCOS may increase the risk of persistent sad mood (78, 79), which may partly explain the higher centrality of sad mood in the present network. Moreover, as a bridging symptom, “sad mood” showed significant associations with “nervousness” (GAD1) and “feeling afraid” (GAD7) within the anxiety symptom cluster. The additional association with “feeling afraid,” which had been less frequently reported, suggests that symptom interrelations may present certain population-specific features in women with PCOS and warrants further investigation. Given evidence that sad mood represents a shared vulnerability factor for comorbid anxiety and depressive disorders (80), it may function as a key emotional linkage within the psychological symptom network of PCOS. In clinical practice, this suggests that addressing and intervening in sad mood may alleviate patients’ broader emotional distress during early psychological management.

The bridge expected influence analysis helps to pinpoint key symptoms that facilitate the emergence and persistence of comorbid psychiatric conditions. In the present study, in addition to “sad mood,” “daytime dysfunction” (PSQI7) and “low energy” (PHQ4) emerged as the most significant bridge symptoms within the network. Sleep problems can generally be categorized into two dimensions: nighttime disturbances and daytime consequences, both of which lead to subjective dissatisfaction with sleep quantity or quality. Daytime dysfunction as a key manifestation of sleep problems (81), has previously been identified as a bridging symptom between sleep problems and emotional symptoms in network studies (65). Low energy typically manifests as persistent fatigue or physical exhaustion, serving as one of the core features of depression (82). Research indicates that both mental disorders and somatic complaints may jointly contribute to fatigue (83), suggesting that chronic psychological distress may translate into somatic symptoms such as reduced energy. Previous network research on individuals with depressive symptoms identified “low energy” as a key bridge symptom between insomnia and depression (84), further supporting the conclusions of this study. In summary, although depression and sleep symptoms exhibit distinct patterns within the network, the reciprocal relationship between “daytime dysfunction” and “low energy” may represent a cross-diagnostic link between them (85). Therefore, in clinical assessment, attention to these two bridge symptoms (daytime dysfunction and low energy) may aid in the earlier identification of PCOS women at elevated risk for psychological comorbidity. However, whether interventions targeting such bridge symptoms can effectively attenuate symptoms or prevent comorbidity requires future interventional studies.

### 4.3 Comparisons based on marital and weight status

Network comparisons based on clinical features (infertility, hirsutism, acne, acanthosis nigricans, and weight status) showed no significant differences between subgroups in overall network strength, structural integrity, or edge weight intensity. These findings suggest that although prior research has demonstrated associations between these clinical characteristics and levels of psychological distress (86), their influence may be insufficient to distinguish the associative patterns among psychological symptoms. A possible explanation is that symptom network analysis focuses on the interactive structure among symptoms rather than the severity of individual symptoms, which may, to some extent, reduce the capacity of traditional clinical features to differentiate between network structures. Furthermore, the incomplete correspondence between biochemical abnormalities and clinical manifestations (e.g., androgen levels versus severity of hirsutism and acne) (87), the time-dependent nature of infertility-related psychological burden (88), and individual differences in psychosocial stress perception and coping may further weaken subgroup-level structural distinctions.

From a clinical perspective, the relatively consistent network structure across clinical subgroups suggests that the interrelations among anxiety, depression, and sleep problems may represent shared psychological processes in women with PCOS. This observation supports the potential applicability of symptom-focused psychological intervention frameworks across different clinical presentations, though it does not imply complete uniformity in symptom severity or intervention needs across different clinical subgroups. Given the potential influence of factors such as sample distribution and individual differences, further validation through longitudinal designs and biochemical indicators is warranted in future research.

### 4.4 Limitations and prospects

Of course, there are several limitations in our study. First, the cross-sectional design precludes causal inference, and longitudinal studies are needed to examine the dynamic interactions among anxiety, depression, and sleep problems in PCOS patients. Second, our study was conducted among PCOS patients at a tertiary-level hospital in a first-tier city, and the applicability of these findings across different regions, healthcare tiers, and sociocultural contexts remains to be validated. Third, psychological symptoms were assessed using self-report measures, which may introduce reporting bias. Lastly, due to the retrospective nature of the study, detailed PCOS phenotypic classification (e.g., metabolic vs. reproductive subtypes), clinical and physiological severity (e.g., infertility duration, hormonal and metabolic measures), and psychosocial factors (e.g., reproductive stress, coping capacity) were not consistently available, which may have masked psychological heterogeneity across subgroups and constrained interpretation of symptom network differences. Future studies should adopt prospective designs incorporating detailed PCOS phenotyping as well as comprehensive biological and psychosocial measures to better characterize symptom dynamics and heterogeneity in this population. Further research is also warranted to examine the potential relevance of symptom-focused strategies suggested by network analysis, as well as to explore the relationships between psychological symptoms and PCOS clinical and physiological features, which could provide insights for more targeted and effective management strategies.

## 5 Conclusions

In conclusion, this study is the first article investigating the network structure of anxiety, depression, and sleep problems in patients with PCOS. The network analysis identified “trouble relaxing” and “sad mood” as the most central symptoms, whereas “daytime dysfunction” and “low energy” are key bridging symptoms. Notably, a strong association between “worthlessness” and “suicidal ideation” was observed, underscoring the clinical relevance of negative self-evaluation in identifying individuals at elevated psychological risk. Symptoms related to stress and fatigue were particularly prominent, highlighting their central role in psychological distress among women with PCOS. Overall, these findings provide a symptom-level perspective on mental health in women with PCOS and offer guidance for future exploration of assessment and intervention strategies targeting key psychological symptoms.

## Data Availability

The original contributions presented in the study are included in the article/**Supplementary Material**. Further inquiries can be directed to the corresponding author.
