# Supplementary material for: Mapping the network structure of anxiety, depression, and sleep symptoms in patients with polycystic ovary syndrome
Source: Front Psychiatry. 2026 Jan 30;17:1738355. doi: 10.3389/fpsyt.2026.1738355 (PMC12900716; doi:10.3389/fpsyt.2026.1738355)
Supplement: Supplementary file 1 [file Supplementaryfile1.docx]

**Supplementary Material**

Mapping the network structure of anxiety, depression, and sleep symptoms in patients with polycystic ovary syndrome

Table S1. Basic information of scales and Descriptive item statistics

Table S2. Correlation matrix of Anxiety, Depression and Sleep problems

Figure S1. Network stability and accuracy results of Anxiety, Depression and Sleep problems

Figure S2. Comparison of network structures based on weight status

Figure S3. Comparison of network structures based on infertility status

Figure S4. Comparison of network structures based on hirsutism status

Figure S5. Comparison of network structures based on acne status

Figure S6. Comparison of network structures based on acanthosis nigricans status

Table S1. Basic information of scales and descriptive item statistics

| **Scale** | **Symptoms** | **Items** | **M** | **SD** | **Expected influence** | **Bridge expected influence** | **Predictability** |
| --- | --- | --- | --- | --- | --- | --- | --- |
| GAD1 | Nervousness | 1. Feeling nervous, anxious or on edge | 0.92 | 0.89 | 0.892 | 0.198 | 0.527 |
| GAD2 | Uncontrollable worry | 2. Not being able to stop or control worrying | 0.66 | 0.84 | 1.064 | 0.158 | 0.592 |
| GAD3 | Excessive worry | 3. Worrying too much about different things | 0.93 | 0.90 | 1.013 | 0.224 | 0.556 |
| GAD4 | Trouble relaxing | 4. Trouble relaxing | 0.65 | 0.84 | 1.142 | 0.386 | 0.576 |
| GAD5 | Restlessness | 5. Being so restless that it is hard to sit still | 0.31 | 0.65 | 0.797 | 0.363 | 0.398 |
| GAD6 | Irritability | 6. Becoming easily annoyed or irritability | 1.02 | 0.91 | 0.963 | 0.295 | 0.484 |
| GAD7 | Feeling afraid | 7. Feeling afraid as if something awful might happen | 0.52 | 0.76 | 0.750 | 0.235 | 0.401 |
| PHQ1 | Anhedonia | 1. Little interest or pleasure in doing things | 0.66 | 0.81 | 0.867 | 0.289 | 0.416 |
| PHQ2 | Sad mood | 2. Feeling down, depressed, or hopeless | 0.58 | 0.73 | 1.077 | 0.506 | 0.548 |
| PHQ4 | Low energy | 4. Feeling tired or having little energy | 0.92 | 0.94 | 1.065 | 0.578 | 0.516 |
| PHQ5 | Abnormal appetite | 1. Poor appetite or overeating | 0.67 | 0.93 | 0.728 | 0.277 | 0.324 |
| PHQ6 | Worthlessness | 1. Feeling bad about yourself or that you are a failure or have let yourself or your family down | 0.44 | 0.75 | 1.038 | 0.256 | 0.501 |
| PHQ7 | Poor concentration | 1. Trouble concentrating on things, such as reading the newspaper or watching television | 0.39 | 0.75 | 0.781 | 0.171 | 0.353 |
| PHQ8 | Abnormal behavior and speech | 1. Moving or speaking so slowly that other people could have noticed? Or the opposite being so fidgety or restless that you have been moving around a lot more than usual | 0.25 | 0.61 | 0.886 | 0.345 | 0.413 |
| PHQ9 | Suicidal ideation | 1. Thoughts that you would be better off dead, or of hurting yourself | 0.13 | 0.44 | 0.525 | 0.086 | 0.261 |
| PSQI1 | Subjective sleep quality | Subjective sleep quality | 1.15 | 0.73 | 1.009 | 0.181 | 0.438 |
| PSQI2 | Sleep latency | Sleep latency | 1.11 | 0.94 | 0.764 | 0.113 | 0.382 |
| PSQI3 | Sleep duration | Sleep duration | 0.97 | 0.81 | 0.566 | 0.099 | 0.211 |
| PSQI4 | Sleep efficiency | Sleep efficiency | 0.42 | 0.79 | 0.418 | -0.004 | 0.183 |
| PSQI5 | Sleep disturbance | Sleep disturbance | 0.80 | 0.57 | 0.812 | 0.290 | 0.327 |
| PSQI6 | Use of sleep medication | Use of sleep medication | 0.05 | 0.31 | 0.189 | 0.062 | 0.062 |
| PSQI7 | Daytime dysfunction | Daytime dysfunction | 1.31 | 0.95 | 0.964 | 0.681 | 0.490 |

Table S2. Correlation matrix of Anxiety, Depression and Sleep problems

|  | GAD1 | GAD2 | GAD3 | GAD4 | GAD5 | GAD6 | GAD7 | PHQ1 | PHQ2 | PHQ4 | PHQ5 | PHQ6 | PHQ7 | PHQ8 | PHQ9 | PSQI1 | PSQI2 | PSQI3 | PSQ4 | PSQI5 | PSQI6 | PSQ7 |
| --- | --- | --- | --- | --- | --- | --- | --- | --- | --- | --- | --- | --- | --- | --- | --- | --- | --- | --- | --- | --- | --- | --- |
| GAD1 | 0.000 | 0 |  |  |  |  |  |  |  |  |  |  |  |  |  |  |  |  |  |  |  |  |
| GAD2 | 0.244 | 0.000 |  |  |  |  |  |  |  |  |  |  |  |  |  |  |  |  |  |  |  |  |
| GAD3 | 0.136 | 0.202 | 0.000 |  |  |  |  |  |  |  |  |  |  |  |  |  |  |  |  |  |  |  |
| GAD4 | 0.123 | 0.216 | 0.159 | 0.000 |  |  |  |  |  |  |  |  |  |  |  |  |  |  |  |  |  |  |
| GAD5 | 0.017 | 0.076 | 0.014 | 0.142 | 0.000 |  |  |  |  |  |  |  |  |  |  |  |  |  |  |  |  |  |
| GAD6 | 0.165 | 0.086 | 0.154 | 0.074 | 0.059 | 0.000 |  |  |  |  |  |  |  |  |  |  |  |  |  |  |  |  |
| GAD7 | 0.010 | 0.082 | 0.125 | 0.043 | 0.127 | 0.129 | 0.000 |  |  |  |  |  |  |  |  |  |  |  |  |  |  |  |
| PHQ1 | 0.000 | 0.046 | 0.000 | 0.000 | 0.026 | 0.034 | 0.000 | 0.000 |  |  |  |  |  |  |  |  |  |  |  |  |  |  |
| PHQ2 | 0.128 | 0.078 | 0.043 | 0.076 | 0.026 | 0.051 | 0.084 | 0.218 | 0.000 |  |  |  |  |  |  |  |  |  |  |  |  |  |
| PHQ4 | 0.000 | 0.000 | 0.018 | 0.048 | 0.000 | 0.024 | 0.000 | 0.114 | 0.054 | 0.000 |  |  |  |  |  |  |  |  |  |  |  |  |
| PHQ5 | 0.036 | 0.003 | 0.024 | 0.000 | 0.000 | 0.104 | 0.000 | 0.013 | 0.000 | 0.186 | 0.000 |  |  |  |  |  |  |  |  |  |  |  |
| PHQ6 | 0.000 | 0.000 | 0.022 | 0.043 | 0.000 | 0.028 | 0.086 | 0.067 | 0.169 | 0.027 | 0.088 | 0.000 |  |  |  |  |  |  |  |  |  |  |
| PHQ7 | 0.000 | 0.000 | 0.000 | 0.011 | 0.107 | 0.000 | 0.000 | 0.141 | 0.000 | 0.060 | 0.072 | 0.122 | 0.000 |  |  |  |  |  |  |  |  |  |
| PHQ8 | 0.000 | 0.017 | 0.000 | 0.019 | 0.148 | 0.000 | 0.078 | 0.025 | 0.049 | 0.040 | 0.057 | 0.073 | 0.215 | 0.000 |  |  |  |  |  |  |  |  |
| PHQ9 | 0.000 | 0.000 | 0.027 | 0.037 | 0.000 | 0.000 | 0.000 | 0.000 | 0.081 | 0.005 | 0.034 | 0.237 | 0.000 | 0.082 | 0.000 |  |  |  |  |  |  |  |
| PSQI1 | 0.016 | 0.013 | 0.032 | 0.017 | 0.018 | 0.000 | 0.000 | 0.013 | 0.000 | 0.072 | 0.000 | 0.000 | 0.000 | 0.000 | 0.000 | 0.000 |  |  |  |  |  |  |
| PSQI2 | 0.000 | 0.000 | 0.025 | 0.049 | 0.000 | 0.000 | 0.000 | 0.016 | 0.000 | 0.000 | 0.009 | 0.014 | 0.000 | 0.000 | 0.000 | 0.396 | 0.000 |  |  |  |  |  |
| PSQI3 | 0.000 | 0.000 | 0.000 | 0.047 | 0.000 | 0.000 | 0.000 | 0.000 | 0.000 | 0.012 | 0.041 | 0.000 | 0.000 | 0.000 | 0.000 | 0.079 | 0.000 | 0.000 |  |  |  |  |
| PSQI4 | 0.000 | 0.000 | 0.000 | 0.000 | 0.011 | 0.000 | -0.020 | 0.000 | -0.007 | 0.000 | 0.000 | 0.000 | 0.000 | 0.000 | 0.013 | 0.047 | 0.045 | 0.329 | 0.000 |  |  |  |
| PSQI5 | 0.015 | 0.000 | 0.033 | 0.032 | 0.008 | 0.010 | -0.020 | 0.017 | 0.021 | 0.000 | 0.047 | 0.063 | 0.011 | 0.017 | 0.009 | 0.158 | 0.191 | 0.000 | 0.000 | 0.000 |  |  |
| PSQI6 | 0.000 | 0.000 | 0.000 | 0.000 | 0.019 | 0.000 | 0.000 | 0.000 | 0.000 | 0.000 | 0.000 | 0.000 | 0.015 | 0.028 | 0.000 | 0.081 | 0.019 | 0.000 | 0.000 | 0.021 | 0.000 |  |
| PSQI7 | 0.003 | 0.000 | 0.000 | 0.008 | 0.000 | 0.043 | 0.000 | 0.138 | 0.006 | 0.403 | 0.014 | 0.000 | 0.027 | 0.038 | 0.000 | 0.067 | 0.000 | 0.058 | 0.000 | 0.151 | 0.007 | 0.000 |

Figure S1. Network stability and accuracy results of Anxiety, Depression and Sleep problems

| **A:** | **B:** |
| --- | --- |
| 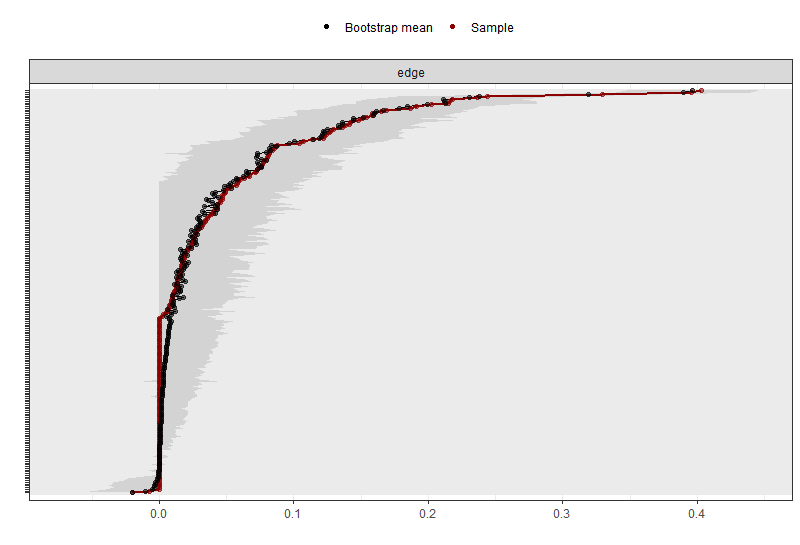 | 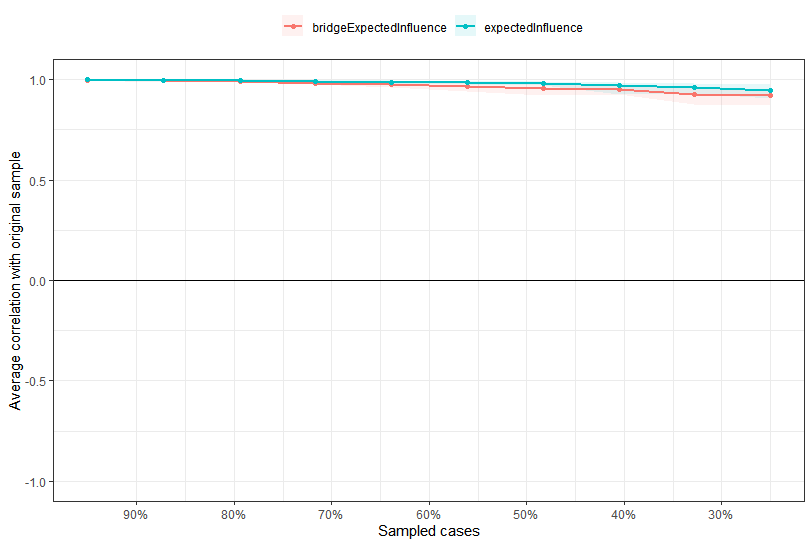 |
| C: | D: |
| 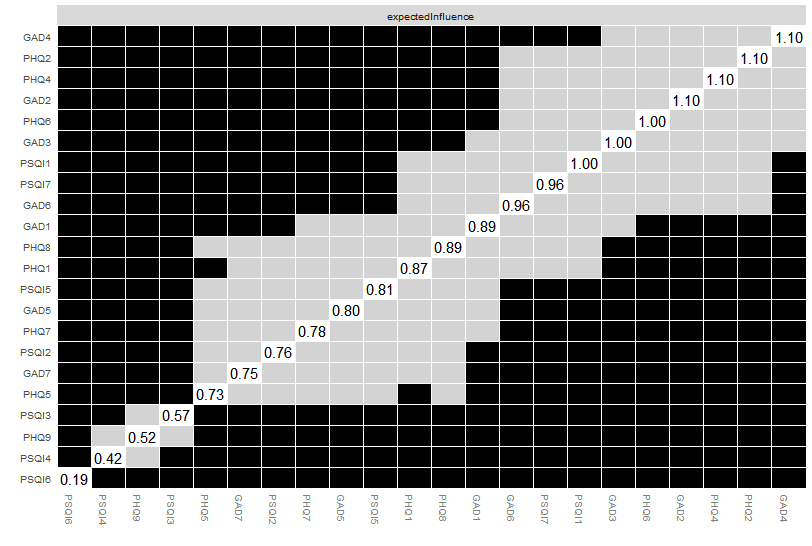 | 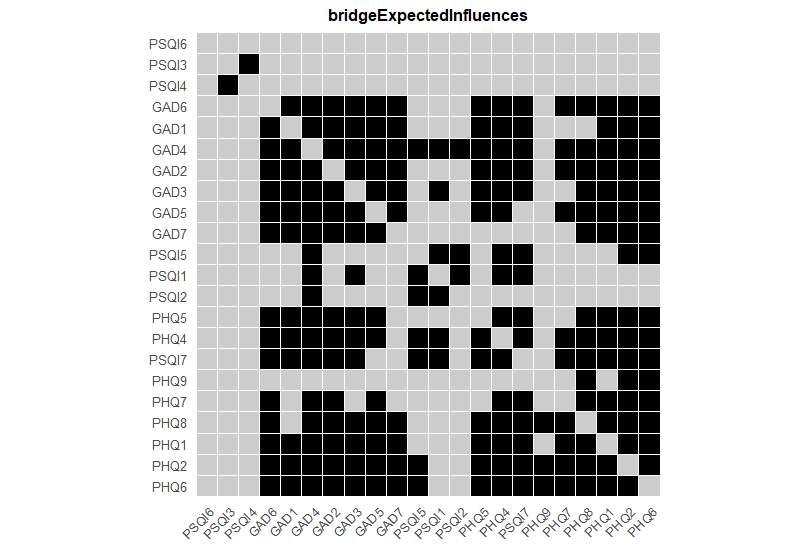 |

| **E:** |
| --- |
| **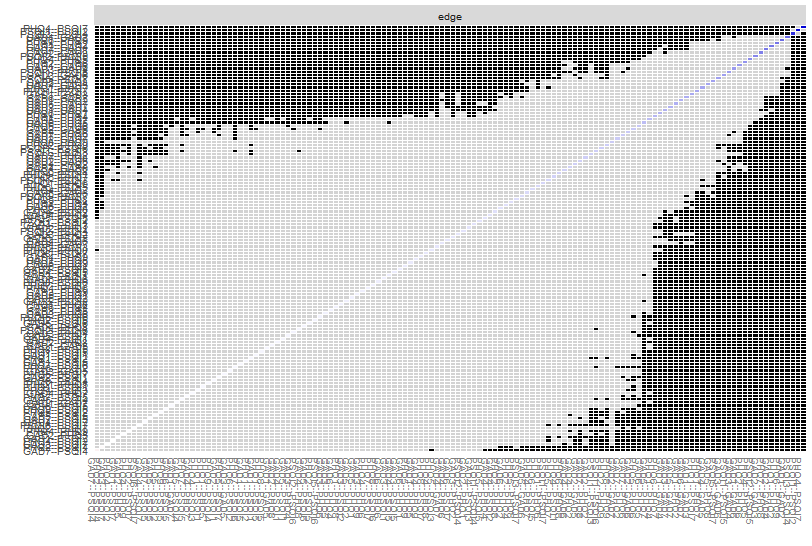** |

Note: (A) Bootstrapped 95% confidence intervals of estimated edges. The red line represents the edge, as estimated in the sample. The grey indicates 95% bootstrapped confidence interval. The x-axis represents the edges, while specific edges are denoted along the y-axis by the grey lines.

(B) Post-hoc stability analysis of node expected influence and bridge expected influence. The x-axis represents the percentage of cases of the original sample used at each step. The y-axis represents the average of correlations between the centrality metrics in the original network and the centrality metrics from the re-estimated networks after excluding increasing percentages of cases.

(C) Nonparametric bootstrapped difference test for expected influence. (D) Nonparametric bootstrapped difference test for bridge expected influence. (E) Nonparametric bootstrapped difference test for edge. Grey boxes indicate no significant difference, whereas black boxes indicate a statistically significant difference.

Figure S2. Comparison of network structures based on weight status

| **A:** | **B:** |
| --- | --- |
| 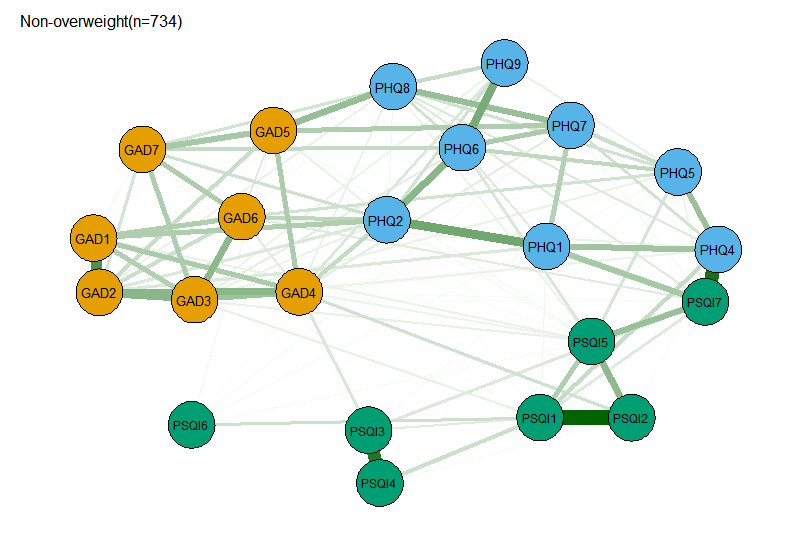 | 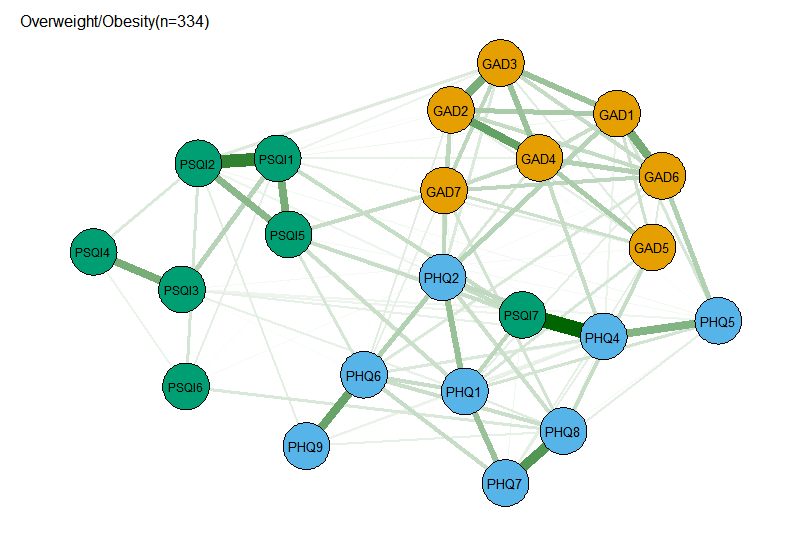 |
| **C:** | **D:** |
| 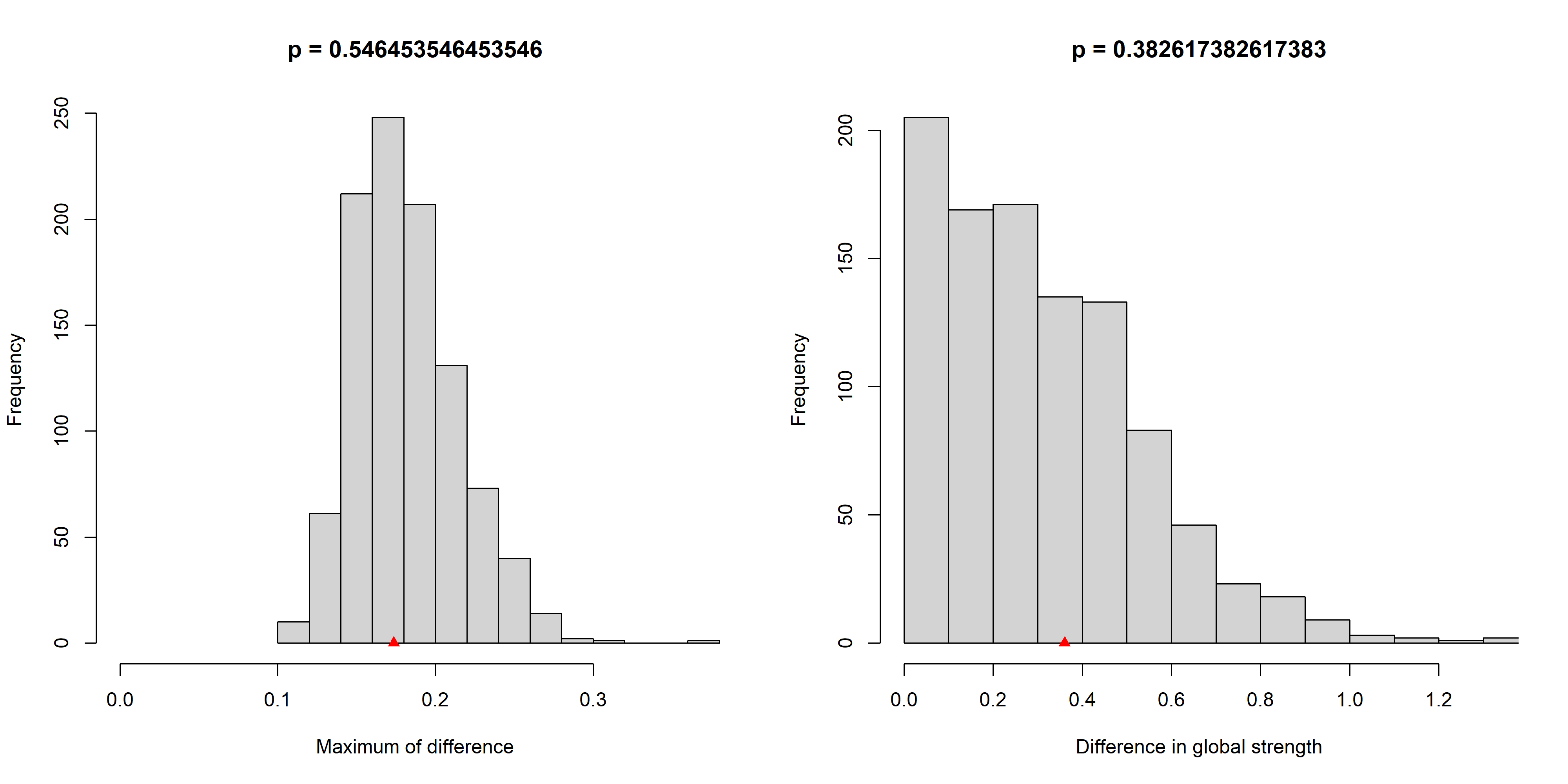 | 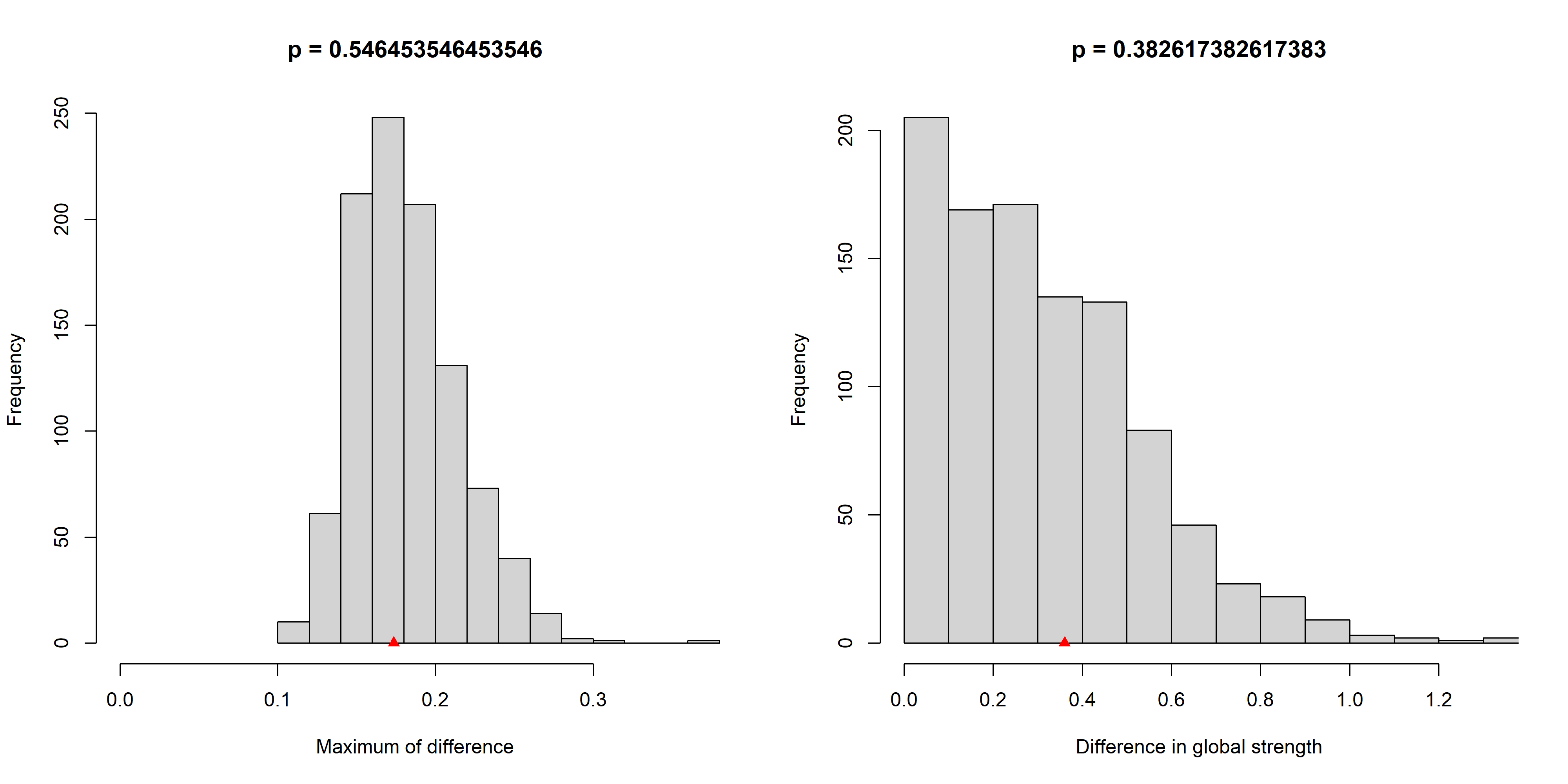 |

Note: (A) Estimated network model in Non-overweight (n=734). (B) Estimated network model in Overweight/obese groups (n=334). (C) A plot of bootstrap value of the difference in network structure. The difference was not significant (M=0.17, *P*=0.546). (D) A plot of bootstrap value of the difference in global strength. The difference was not significant (global strength among Non-overweight: 9.42; among Overweight/obese groups: 9.07; *P*= 0.383).

Figure S3. Comparison of network structures based on infertility status

| **A:** | **B:** |
| --- | --- |
| 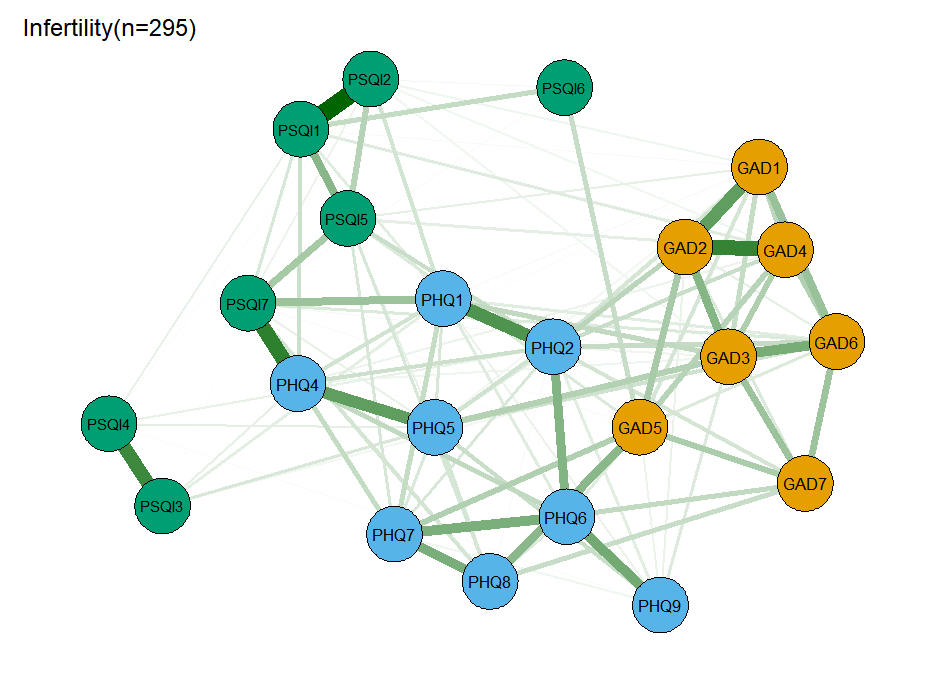 | 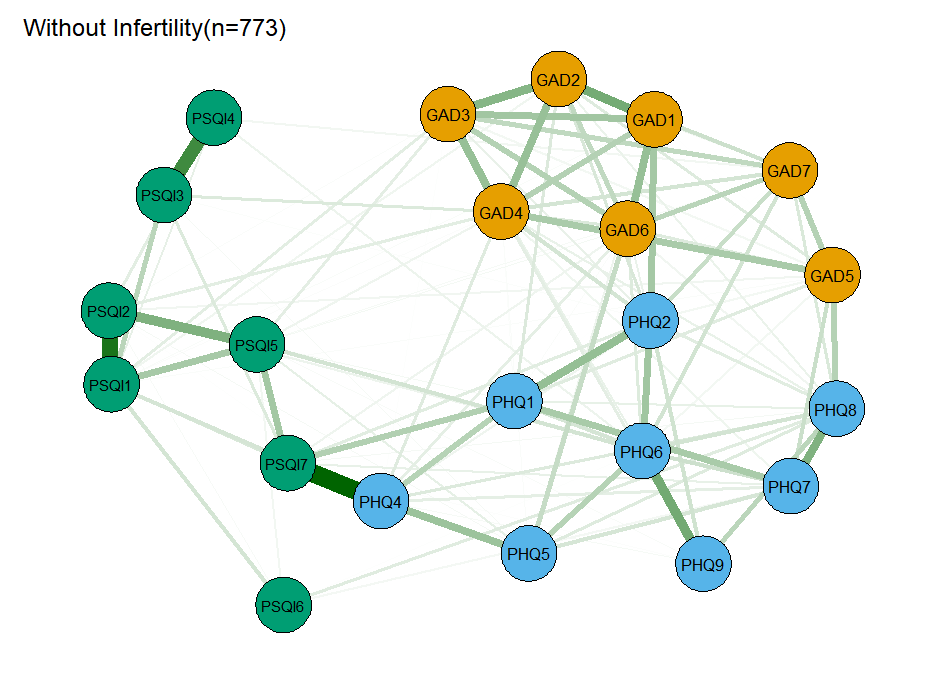 |
| **C:** | **D:** |
| 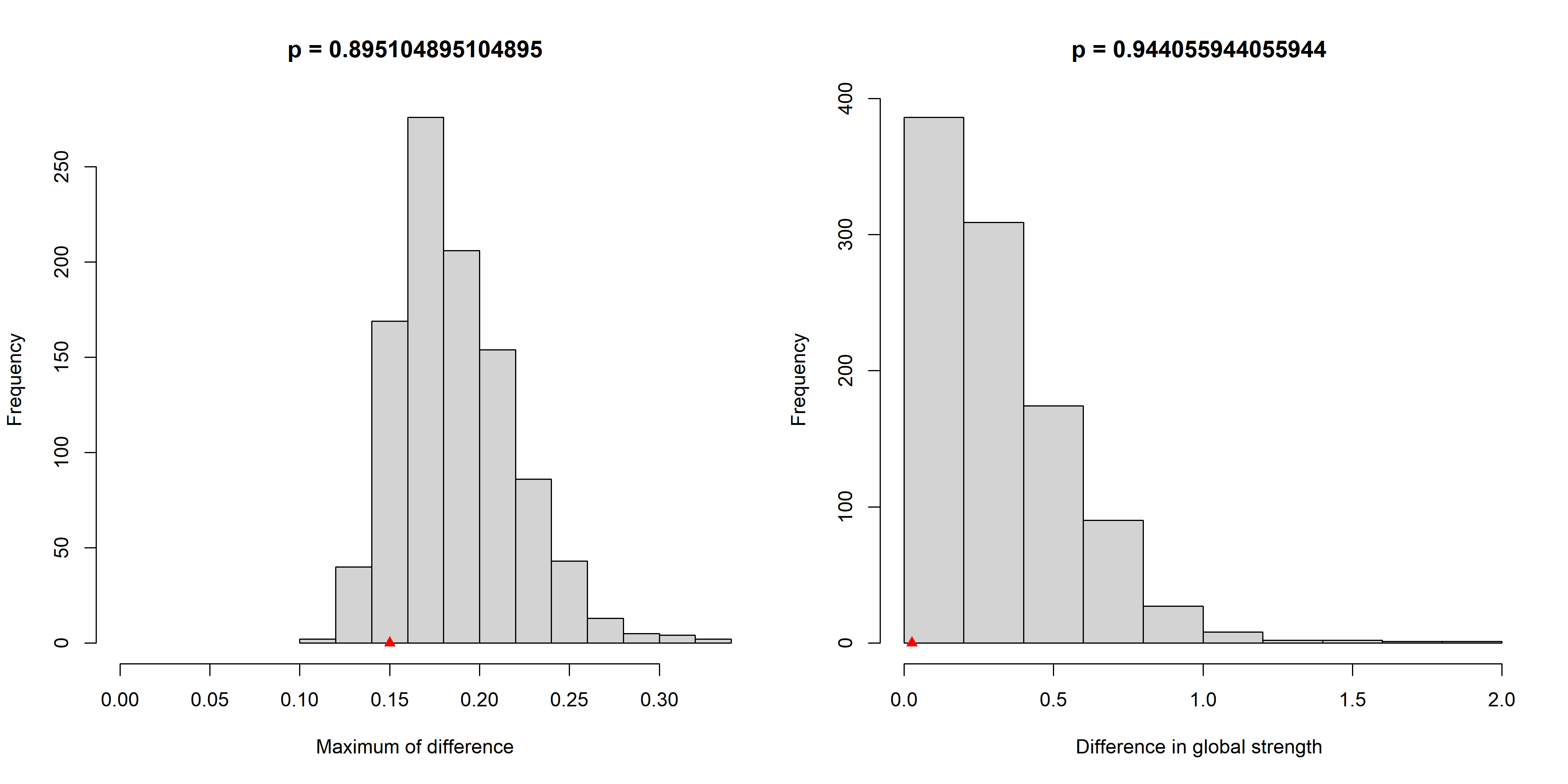 | 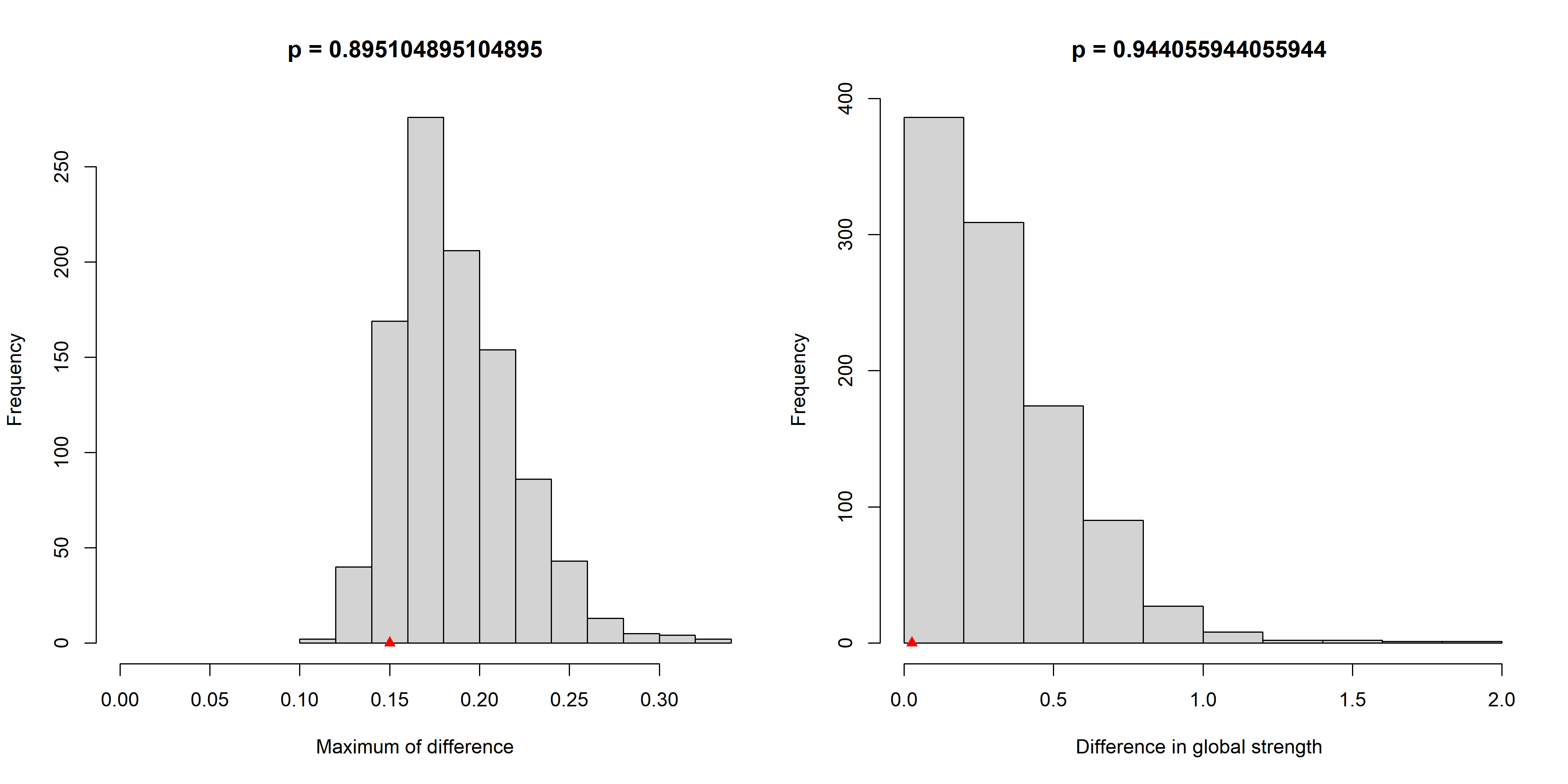 |

Note: (A) Estimated network model in Infertility (n=295). (B) Estimated network model in Without Infertility (n=773). (C) A plot of bootstrap value of the difference in network structure. The difference was not significant (M=0.15, *P*=0.873). (D) A plot of bootstrap value of the difference in global strength. The difference was not significant (global strength among Infertility: 9.25; among Without Infertility groups: 9.28; *P*= 0.950).

Figure S3. Comparison of network structures based on hirsutism status

| **A:** | **B:** |
| --- | --- |
| 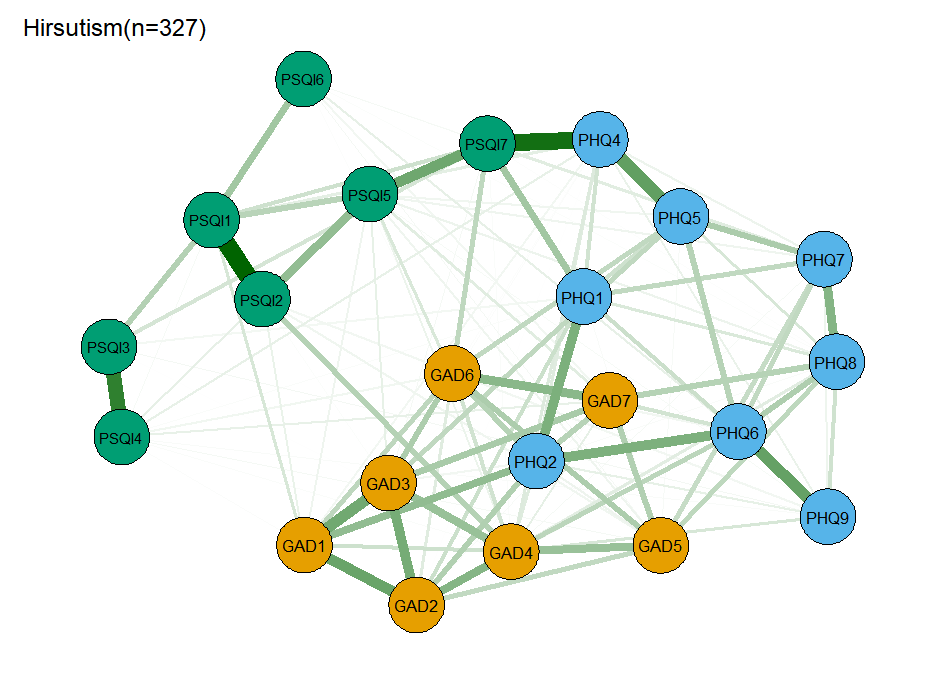 | 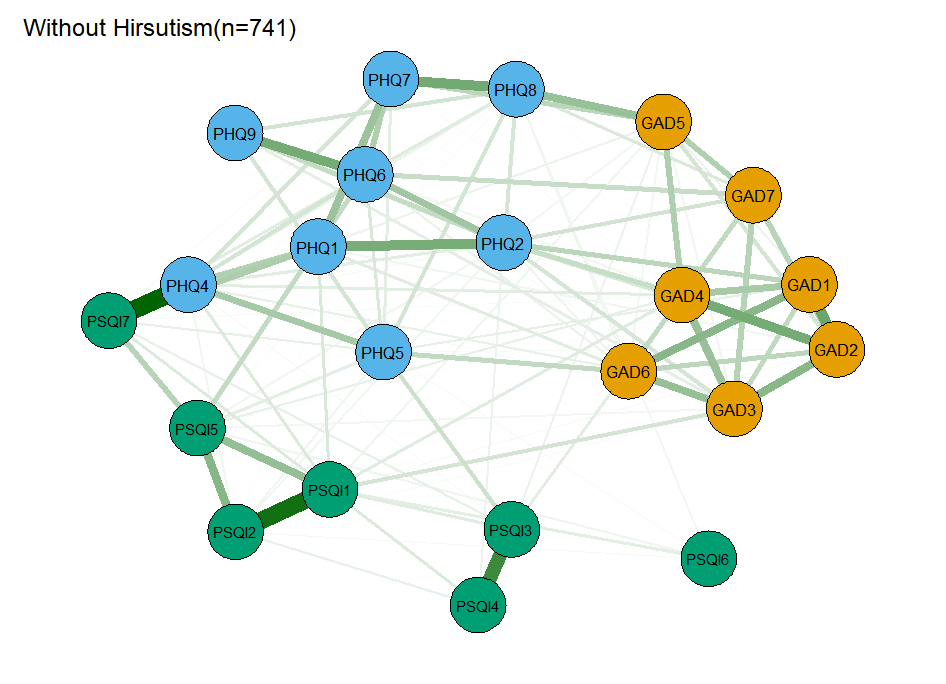 |
| **C:** | **D:** |
| 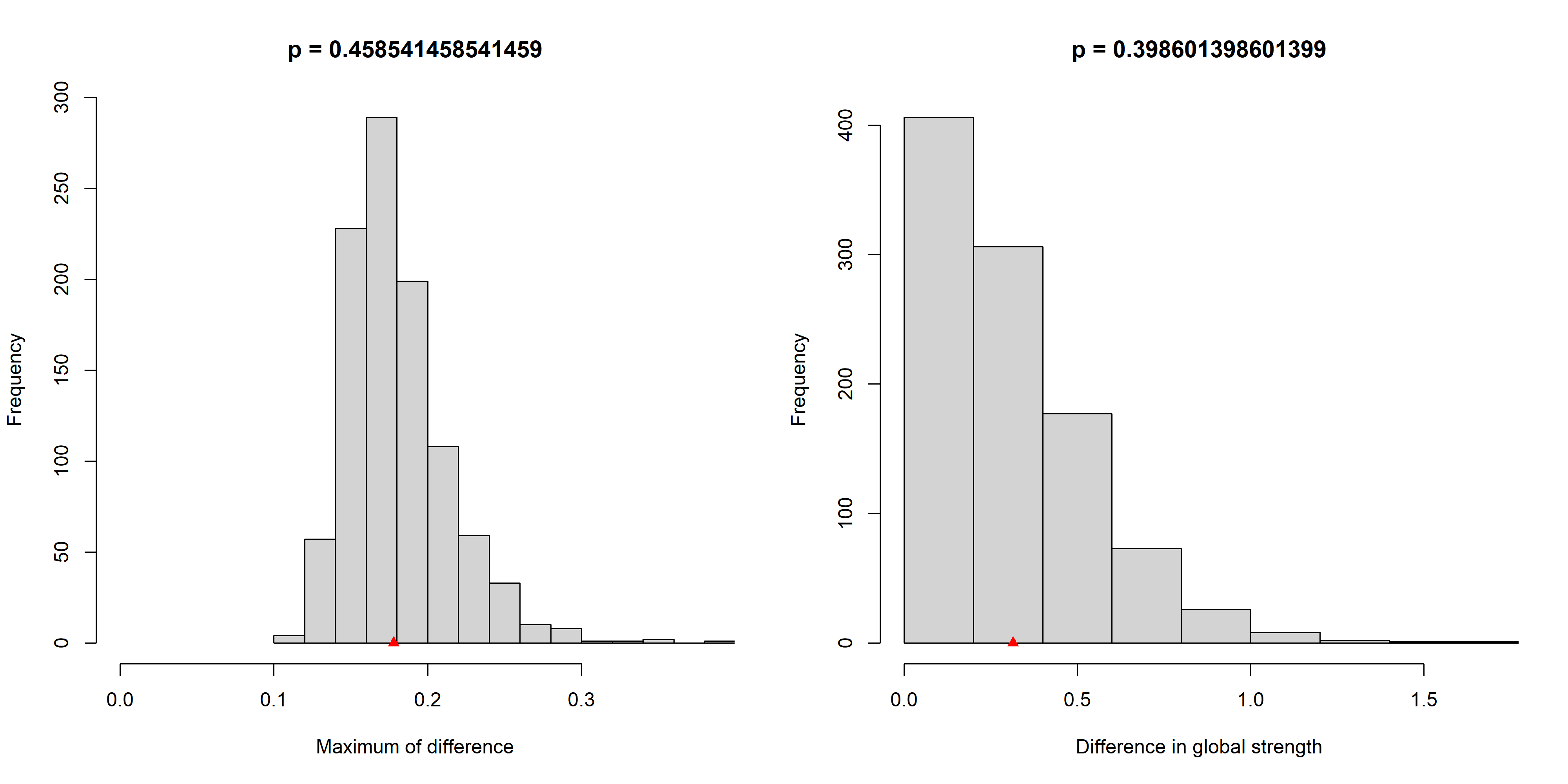 | 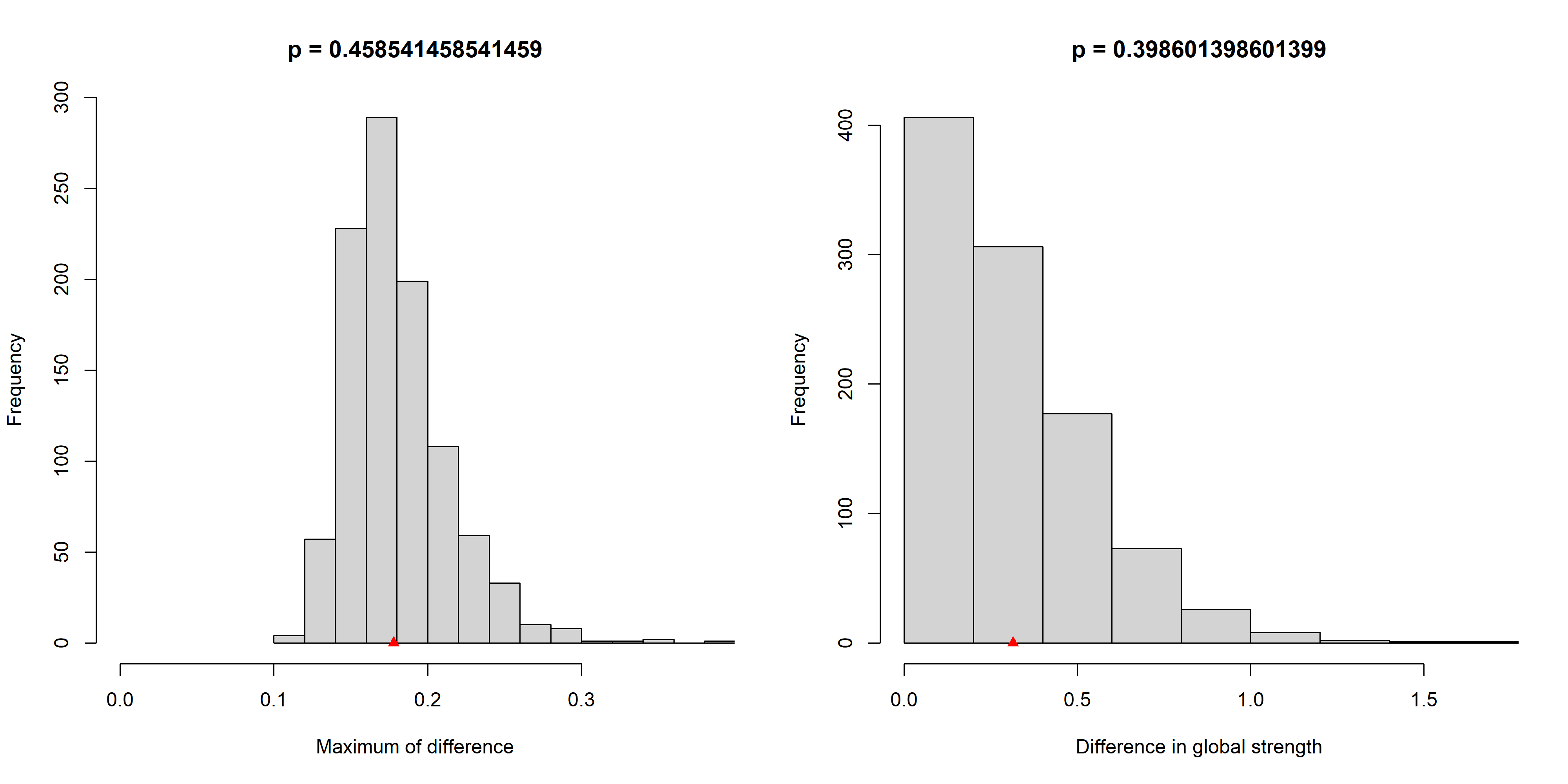 |

Note: (A) Estimated network model in Hirsutism (n=327). (B) Estimated network model in Without Hirsutism (n =741). (C) A plot of bootstrap value of the difference in network structure. The difference was not significant (M=0.18, *P*=0.480). (D) A plot of bootstrap value of the difference in global strength. The difference was not significant (global strength among Hirsutism: 9.61; among Without Hirsutism groups: 9.30; *P*= 0.419).

Figure S4. Comparison of network structures based on acne status

| **A:** | **B:** |
| --- | --- |
| 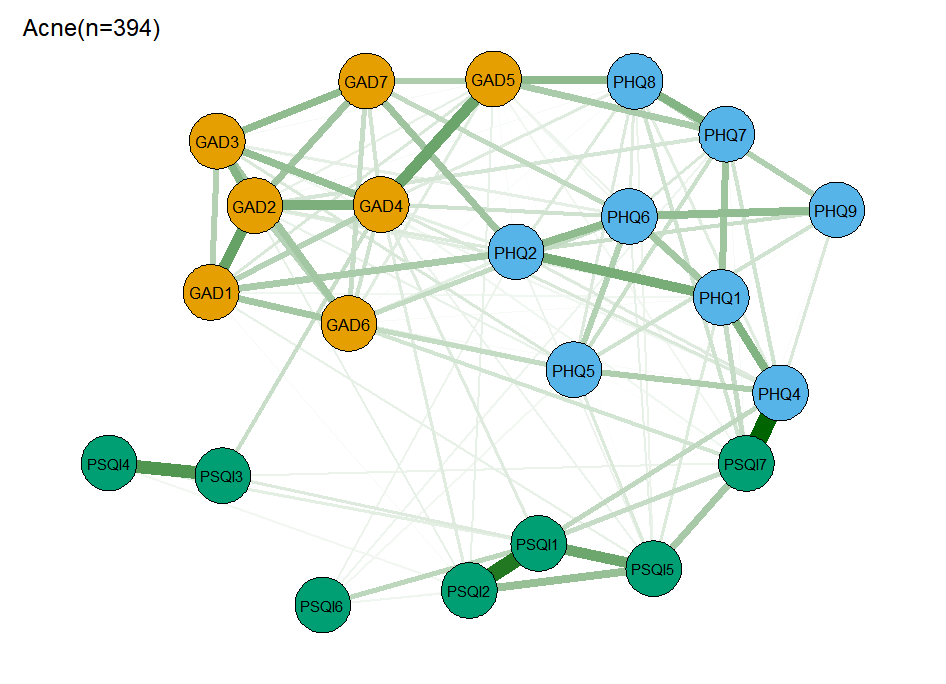 | 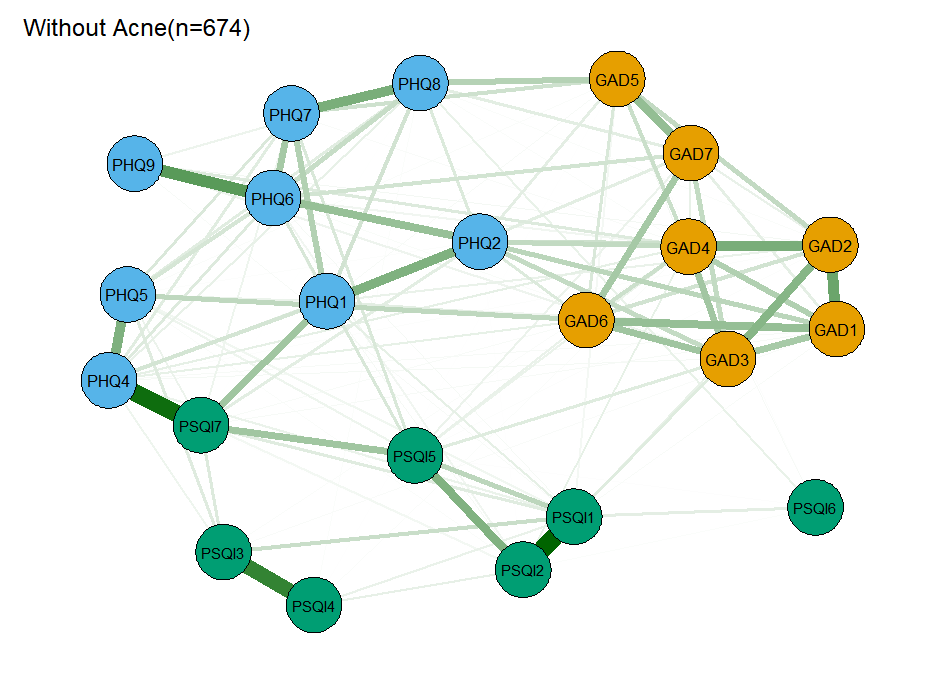 |
| **C:** | **D:** |
| 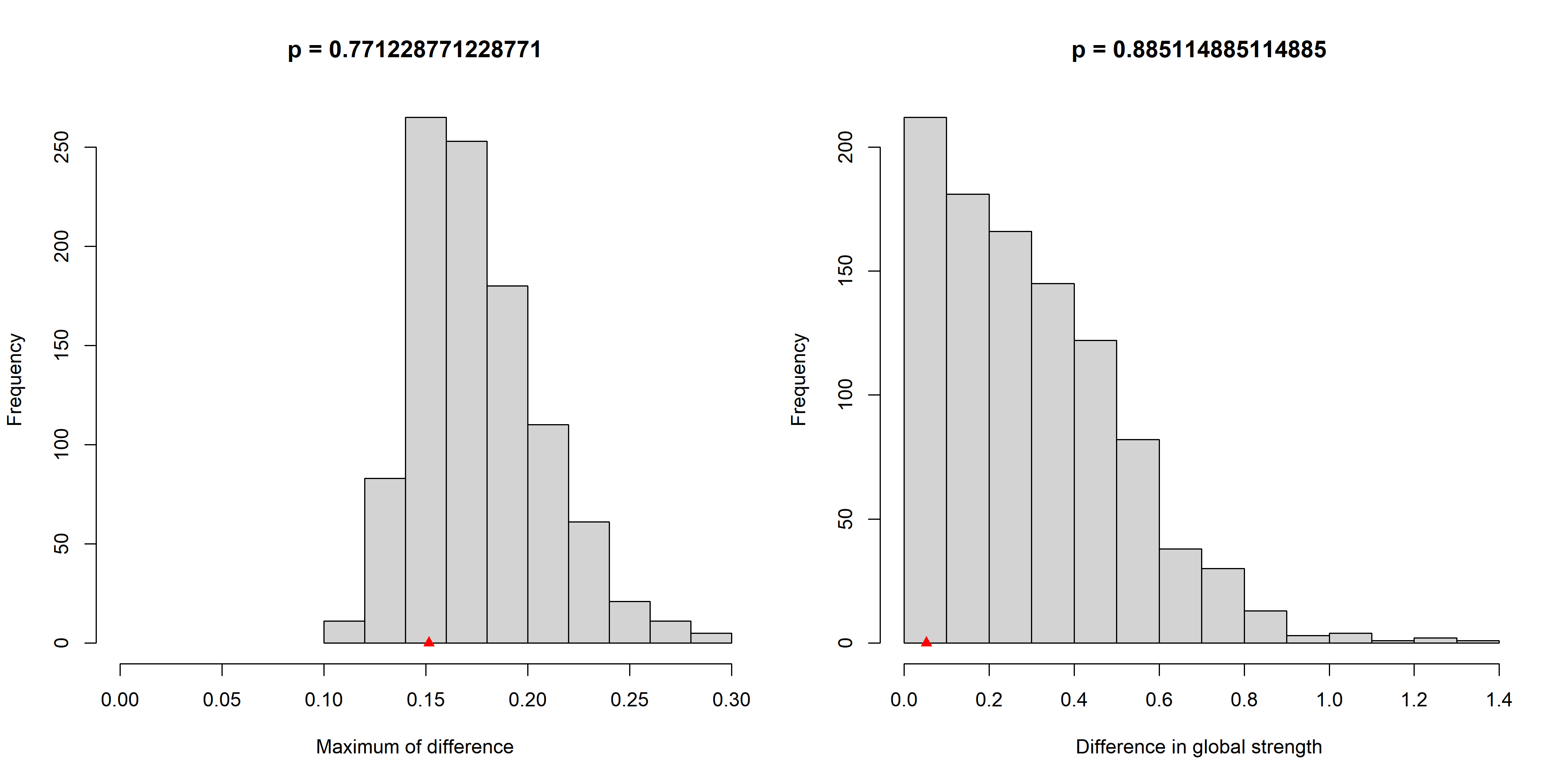 | 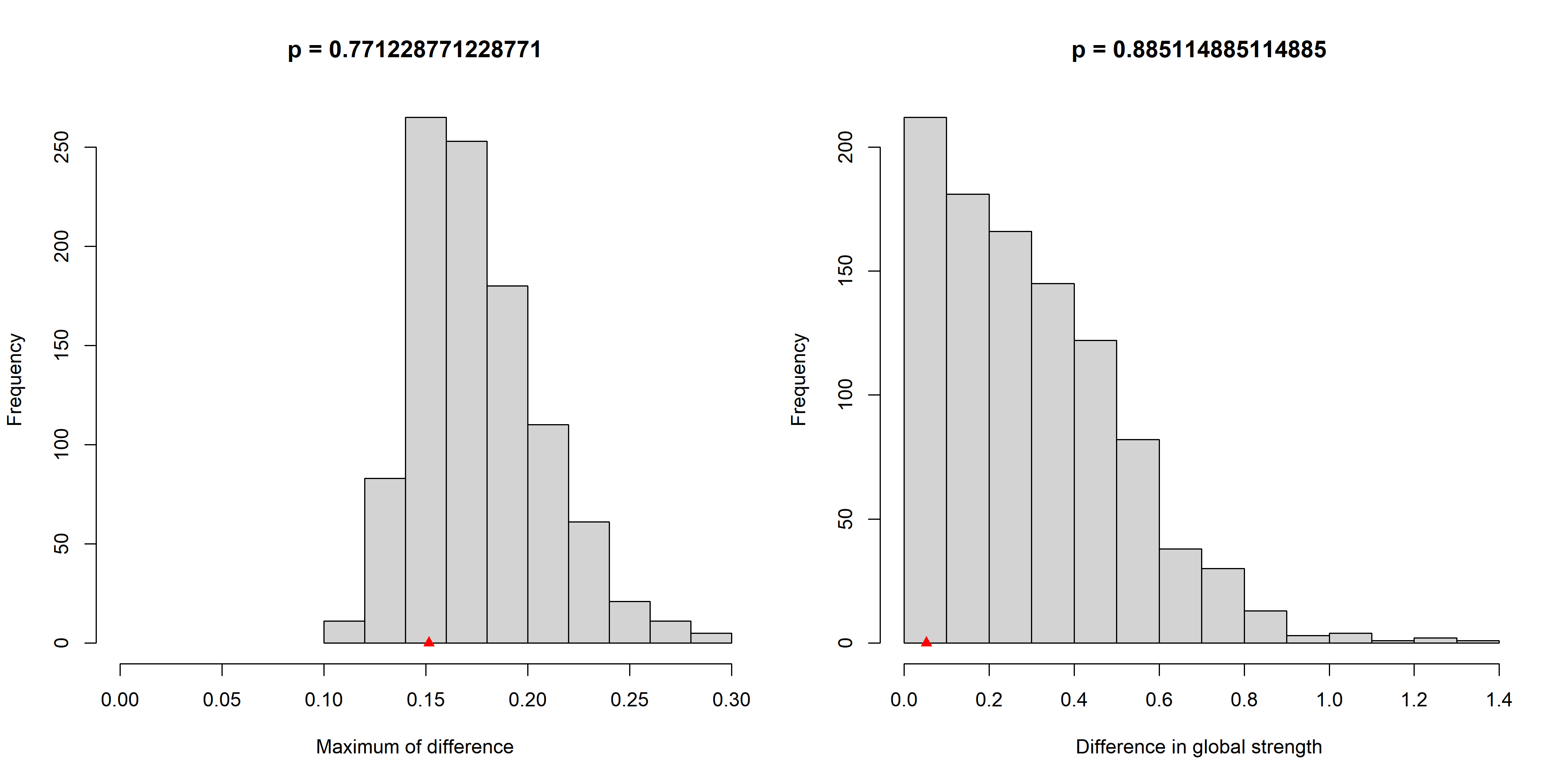 |

Note: (A) Estimated network model in Acne (n=394). (B) Estimated network model in Without Acne (n=674). (C) A plot of bootstrap value of the difference in network structure. The difference was not significant (M=0.15, *P*=0.778). (D) A plot of bootstrap value of the difference in global strength. The difference was not significant (global strength among Acne: 9.15; among Without Acne groups:9.10; *P*= 0.882).

Figure S5. Comparison of network structures based on acanthosis nigricans status

| **A:** | **B:** |
| --- | --- |
| 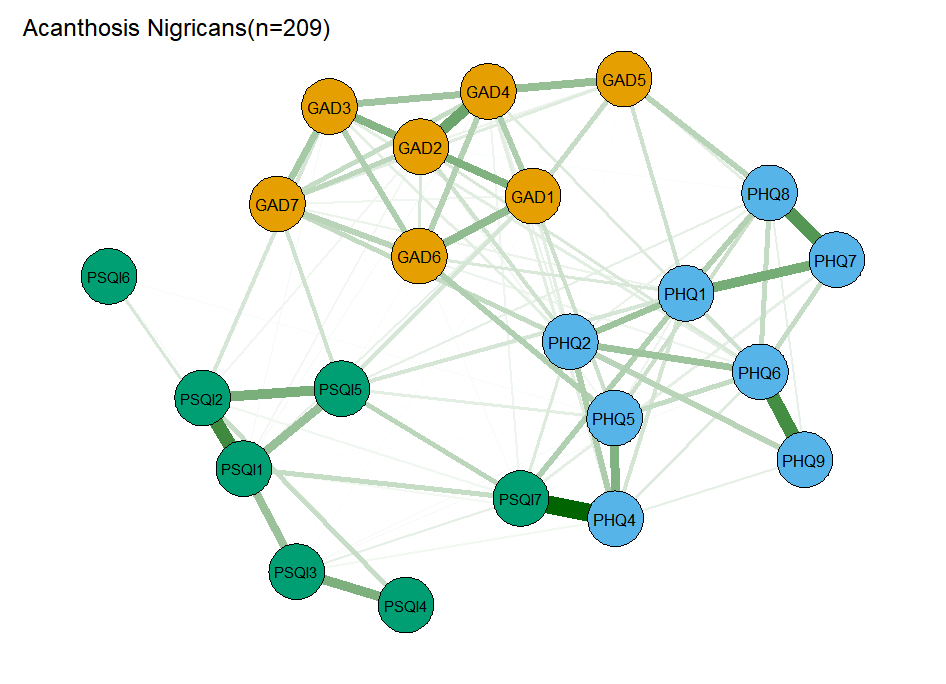 | 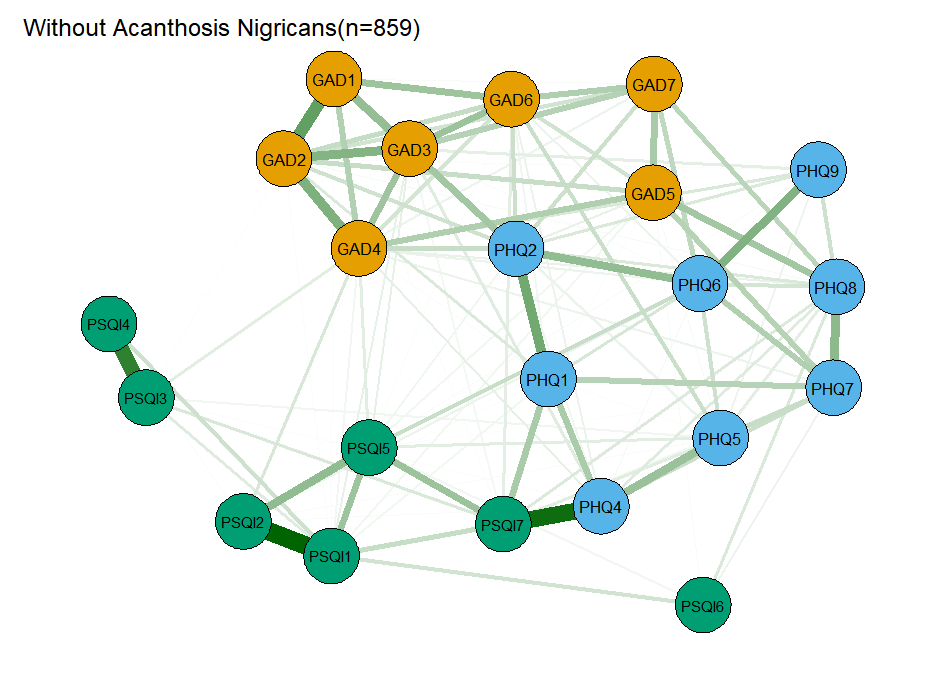 |
| **C:** | **D:** |
| 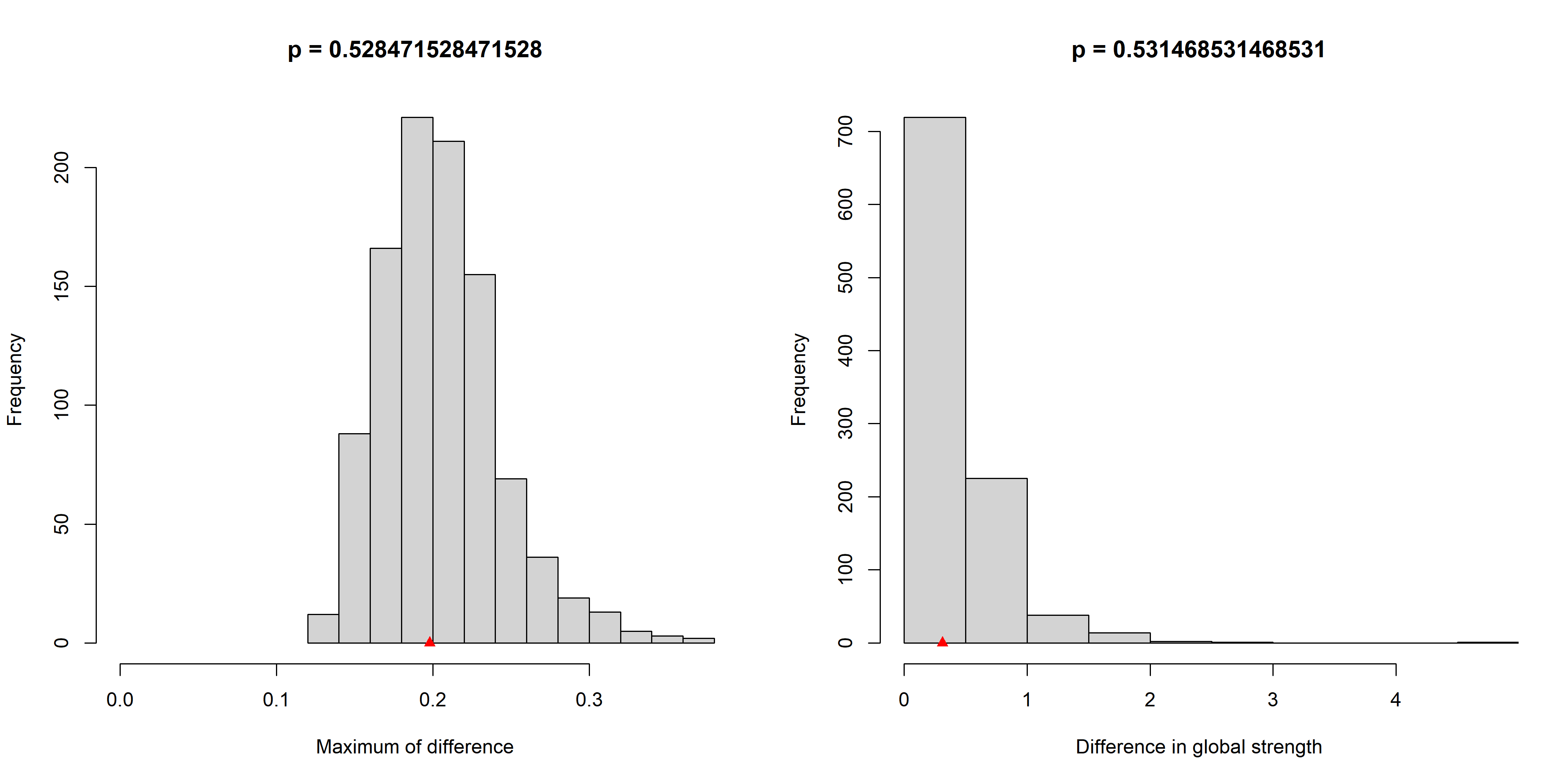 | 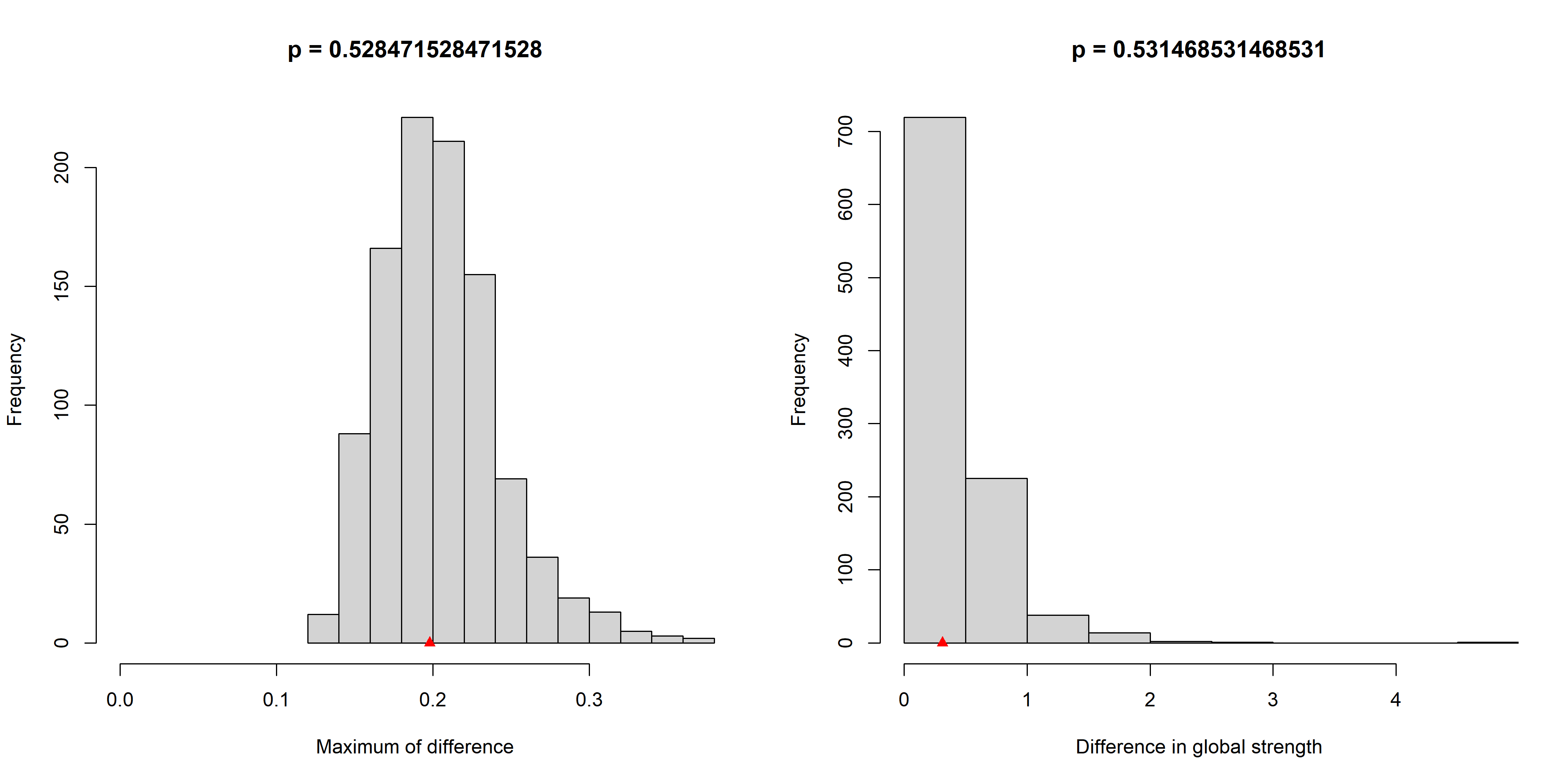 |

Note: (A) Estimated network model in Acanthosis Nigricans (n=209). (B) Estimated network model in Without Acanthosis Nigricans (n =859). (C) A plot of bootstrap value of the difference in network structure. The difference was not significant (M=0.20, *P*=0.528). (D) A plot of bootstrap value of the difference in global strength. The difference was not significant (global strength among Acanthosis Nigricans: 9.04; among Without Acanthosis Nigricans:9.35; *P*=0.531).
